# Spatially dispersed synapses yield sharply‐tuned place cell responses through dendritic spike initiation

**DOI:** 10.1113/JP275310

**Published:** 2018-07-17

**Authors:** Reshma Basak, Rishikesh Narayanan

**Affiliations:** ^1^ Cellular Neurophysiology Laboratory Molecular Biophysics Unit Indian Institute of Science Bangalore India

**Keywords:** active dendrites, computational model, dendritic spikes, hippocampus, place cells, synaptic clustering

## Abstract

**Key points:**

- The generation of dendritic spikes and the consequent sharp tuning of neuronal responses are together attainable even when iso‐feature synapses are randomly dispersed across the dendritic arbor.
- Disparate combinations of channel conductances with distinct configurations of randomly dispersed place field synapses concomitantly yield similar sharp tuning profiles and similar functional maps of several intrinsic properties.
- Targeted synaptic plasticity converts silent cells to place cells for specific place fields in models with disparate channel combinations that receive dispersed synaptic inputs from multiple place field locations.
- Dispersed localization of iso‐feature synapses is a strong candidate for achieving sharp feature selectivity in neurons across sensory‐perceptual systems, with several degrees of freedom in relation to synaptic locations.
- Quantitative evidence for the possibility that degeneracy (i.e. the ability of disparate structural components to yield similar functional outcomes) could act as a broad framework that effectively accomplishes the twin goals of input‐feature encoding and homeostasis of intrinsic properties without cross interferences.

**Abstract:**

A prominent hypothesis spanning several sensory‐perceptual systems implicates spatially clustered synapses in the generation of dendritic spikes that mediate sharply‐tuned neuronal responses to input features. In this conductance‐based morphologically‐precise computational study, we tested this hypothesis by systematically analysing the impact of distinct synaptic and channel localization profiles on sharpness of spatial tuning in hippocampal pyramidal neurons. We found that the generation of dendritic spikes, the emergence of an excitatory ramp in somatic voltage responses, the expression of several intrinsic somatodendritic functional maps and sharp tuning of place‐cell responses were all attainable even when iso‐feature synapses are randomly dispersed across the dendritic arbor of models with disparate channel combinations. Strikingly, the generation and propagation of dendritic spikes, reliant on dendritic sodium channels and *N*‐methyl‐d‐asparate receptors, mediated the sharpness of spatial tuning achieved with dispersed synaptic localization. To ensure that our results were not artefacts of narrow parametric choices, we confirmed these conclusions with independent multiparametric stochastic search algorithms spanning thousands of unique models for each synaptic localization scenario. Next, employing virtual knockout models, we demonstrated a vital role for dendritically expressed voltage‐gated ion channels, especially the transient potassium channels, in maintaining sharpness of place‐cell tuning. Importantly, we established that synaptic potentiation targeted to afferents from one specific place field was sufficient to impart place field selectivity even when intrinsically disparate neurons received randomly dispersed afferents from multiple place field locations. Our results provide quantitative evidence for disparate combinations of channel and synaptic localization profiles to concomitantly yield similar tuning and similar intrinsic properties.

## Key points

- The generation of dendritic spikes and the consequent sharp tuning of neuronal responses are together attainable even when iso‐feature synapses are randomly dispersed across the dendritic arbor.
- Disparate combinations of channel conductances with distinct configurations of randomly dispersed place field synapses concomitantly yield similar sharp tuning profiles and similar functional maps of several intrinsic properties.
- Targeted synaptic plasticity converts silent cells to place cells for specific place fields in models with disparate channel combinations that receive dispersed synaptic inputs from multiple place field locations.
- Dispersed localization of iso‐feature synapses is a strong candidate for achieving sharp feature selectivity in neurons across sensory‐perceptual systems, with several degrees of freedom in relation to synaptic locations.
- Quantitative evidence for the possibility that degeneracy (i.e. the ability of disparate structural components to yield similar functional outcomes) could act as a broad framework that effectively accomplishes the twin goals of input‐feature encoding and homeostasis of intrinsic properties without cross interferences.

## Introduction

A prominent hypothesis spanning several perceptual systems implicates spatially clustered synapses in the generation of dendritic spikes (dSpikes) that mediate sharply‐tuned neuronal responses to input features (Losonczy *et al*. 2008; Govindarajan *et al*. 2011; Makino & Malinow, 2011; Takahashi *et al*. 2012; DeBello *et al*. 2014; Druckmann *et al*. 2014; Wilson *et al*. 2016). On the other hand, there are significant lines of evidence, spanning several perceptual systems, for similarly‐tuned synaptic inputs to be dispersed across the dendritic arbor (Jia *et al*. 2010; Chen *et al*. 2011; Varga *et al*. 2011; Hill *et al*. 2013; Grienberger *et al*. 2015; Domnisoru & Tank, 2016, 2017). How do we reconcile these apparently contradictory observations where spatial clustering is postulated to be required for eliciting dSpikes and synaptic localization is shown to be dispersed? Could lines of evidence that dSpikes could be elicited when several spatially distributed synapses are activated (Cannon *et al*. 2010) provide a reconciliation with reference to sharpness of tuning properties that might be attained with specific temporal activation profiles of dispersed afferent inputs?

In this context, a predominant dogma regarding pyramidal cell neurophysiology is that linear or supralinear modes of operation are respectively preferred when synapses are dispersed or clustered, where the supralinear mode of operation recruits dSpikes as a result of cooperativity among concomitantly activated synaptic inputs (Grienberger *et al*. 2015). Is spatial clustering of synapses an essential component for the expression of concomitantly active synaptic inputs towards the generation of dSpikes? Are spatial clustering of synapses and dSpikes essential for achieving sharp tuning to input features? How do the different voltage‐gated ion channels and synaptic receptors that are expressed in the dendritic arbor of pyramidal neurons contribute to sharp tuning to input features? Could similar sharp tuning to input features and similar intrinsic electrophysiological characteristics be obtained with distinct configurations of randomly dispersed synapses in neurons endowed with disparate channel localization profiles?

In this conductance‐based morphologically‐precise computational study, we tested the hypothesis concerning the link between spatially clustered synapses and sharply tuned responses by systematically analysing the impact of distinct synaptic and channel localization profiles on sharpness of spatial tuning in hippocampal pyramidal neurons. We found that sharply‐tuned firing responses were achieved in models where synapses were all clustered on the soma or were randomly dispersed across the dendritic arbor, although not in cases where the same set of synapses were localized to one or two obliques. Strikingly, dSpikes were more prevalent when synapses were randomly dispersed, with sharpness of spatial tuning mediated by the generation and propagation of dSpikes reliant on dendritic sodium channels, transient potassium channels and NMDA receptors. We confirmed these results with independent multiparametric stochastic search algorithms spanning thousands of unique models for each synaptic localization scenario. Our results from these stochastic search algorithms clearly demonstrate the ability of disparate channel conductances with distinct configurations of randomly dispersed place field synapses to concomitantly yield similar sharp tuning profiles and similar functional maps of several intrinsic properties. Finally, we also quantitatively demonstrate that synaptic potentiation targeted to afferents from one specific place field is sufficient to enforce place field selectivity, even when intrinsically disparate neurons received randomly dispersed afferents from multiple place field locations.

Our results provide clear lines of quantitative evidence that spatial clustering of synapses is neither essential for the generation of dSpikes, nor a requirement for sharp tuning of neuronal responses to input features. Our results also present quantitative evidence for degeneracy, the ability of disparate structural components to yield similar functional outcomes (Edelman & Gally, 2001), as a broad framework that could encompass effective input‐feature encoding and homeostasis of intrinsic properties. Specifically, our demonstration that disparate channel combinations and distinctly dispersed synaptic localization could yield similar tuning profiles and similar intrinsic functional maps argues for multiple non‐unique routes with respect to achieving the twin goals of encoding and homeostasis in neurons. We argue that this ability of neurons to achieve non‐linear input processing and sharp feature selectivity with randomly dispersed iso‐feature synapses and disparate channel combinations equips them with significant degrees of freedom towards achieving sharp tuning. These quantitative lines of evidence also dispel the impression that dispersed and clustered synaptic localization strategies exclusively translate to linear and non‐linear modes of dendritic operation, respectively. Taken together, we postulate distinct advantages for the dispersed localization strategy, especially for spatial tuning in the adult hippocampus where new place cells are formed in an experience‐dependent manner.

## Methods

**Figure 1:**
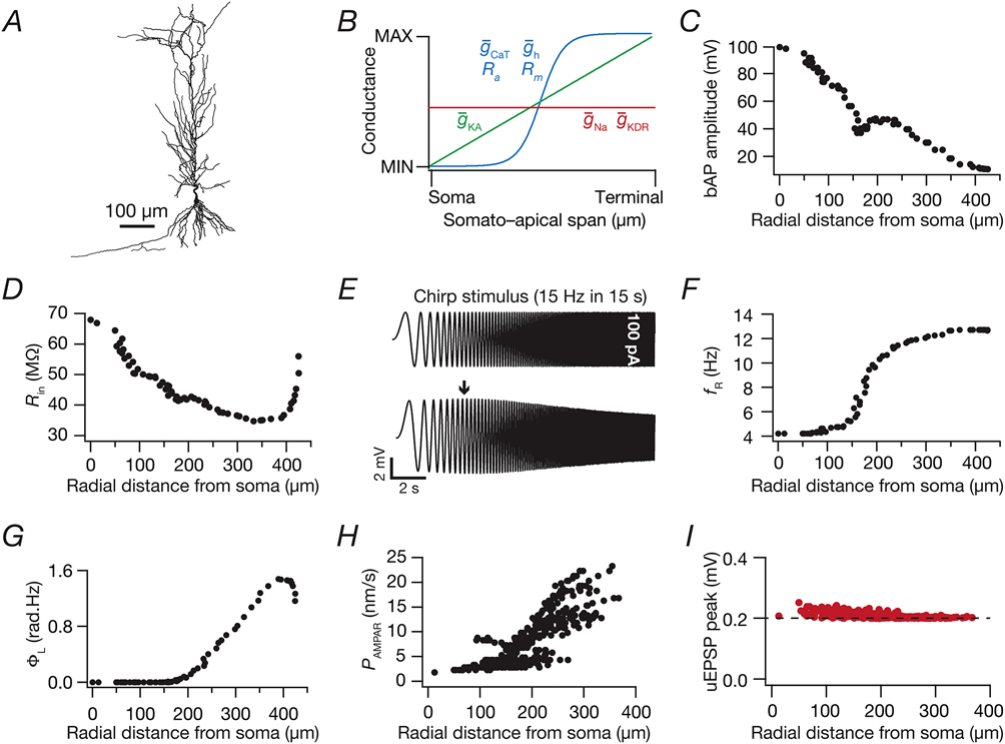
Experimental constraints on the intrinsic and synaptic properties of a morphologically realistic CA1 pyramidal model neuron *A*, 2‐D projection of a 3‐D reconstructed hippocampal CA1 pyramidal neuron model used as the base model. *B*, experimentally constrained somatoapical distributions of active and passive parameters (see Table 1) in the base model adopted from (Rathour and Narayanan, 2014) to match physiological measurements in (*C*) to (*G*). *C*–*G*, bAP amplitude (*C*), input resistance (*R*_in_) (*D*), resonance frequency (*f*_R_) (*F*) and total inductive phase (Φ_L_) (*G*) plotted as a function of radial distance from the soma. A chirp current stimulus, 100 pA in peak‐to‐peak amplitude and frequency linearly increasing from 0.1 Hz to 15 Hz in 15 s (*E*, top) was injected at the soma to record local voltage responses (*E*, bottom). The arrow marks the *f*_R_. *H*, location‐dependent permeability values of AMPA receptor that normalized somatic unitary postsynaptic potential (uEPSP) amplitudes to around 0.2 mV as depicted in (*I*). [Color figure can be viewed at http://wileyonlinelibrary.com]

A morphologically realistic reconstruction of a CA1 neuron (*n123*) was obtained from the NeuroMorpho database (Pyapali *et al*. 1998; Ascoli *et al*. 2007) and passive and active properties for the base model were adopted from an earlier model (Rathour & Narayanan, 2014) that matched several somatodendritic functional maps (Narayanan & Johnston, 2012) through physiologically established channel localization profiles (Fig. 1 *A–G*). The specific membrane capacitance was set uniformly at 1 μF cm^–2^. *R*_m_ and *R*_a_ were set in a gradient along the trunk as a function of the radial distance of the compartment from the soma according to the equations and parametric values in Tables 1 and 2 (Fig. 1 *B*). The basal dendrites and the axonal compartments had somatic *R*_m_ and *R*_a_, and the apical obliques had the same *R*_m_ and *R*_a_ as the trunk compartment from which they originated. The model was compartmentalized according to the *d*_λ_ rule (Carnevale & Hines, 2006) to ensure isopotentiality in each compartment. Specifically, each compartment was smaller than 0.1 × λ_100_, with λ_100_ representing the space constant of the section computed at 100 Hz. The five different ion channels used in the model were Hodgkin–Huxley‐type delayed rectifier potassium (KDR), fast sodium (NaF), *T‐*type calcium (CaT), hyperpolarization‐activated cyclic‐nucleotide‐gated (HCN) non‐specific cation and *A*‐type potassium (KA) channels (Table 1). Currents through the NaF, KDR, KA and HCN channels employed an Ohmic formulation with reversal potentials for Na^+^, K^+^ and *h* channels set at 55, –90 and –30 mV, respectively. The CaT current was modelled using the Goldman–Hodgkin–Katz (GHK) convention (Shah *et al*. 2008).

**Table 1:** Description of somatodendritic gradients in passive properties and channel gradients used in the model

Parameter	Gradient of distribution
Specific membrane resistivity, *R*_m_	Rm(x)=Rm+(Rm-min−Rm-max)1+exp((Rm- hmp −x)/(Rm− hmp −x)Rm−Rm- slope )kΩcm−2
Axial resisitivity, *R*_a_	Ra(x)=Ra+(Ra-min−Ra-max)1+exp((Ra- hmp −x)/(Ra− hmp −x)Ra−Ra- slope )Ω· cm
Maximal conductance of NaF channels	${\bar{g}}_{\mathrm{Na}}$, uniform across the somatodendritic arbor, with the density at the axon initial segment set at 5${\bar{g}}_{\mathrm{Na}}$
Maximal conductance of KDR channels	${\bar{g}}_{\mathrm{KDR}}$, uniform across the somatodendritic arbor
Maximal conductance of KA channels	g KA (x)=g¯ KA (1+g¯ KA - fold 100) mS cm−2
Maximal conductance of *h* channels	gh(x)=g¯h(1+g¯h- fold 1+exp((g¯h- hmp −x)/(g¯h− hmp −x)g¯h−g¯h- slope ))μScm−2
Maximal conductance of CaT channels	g CaT (x)=g¯ CaT (1+g¯ CaT - fold 1+exp((g¯ CaT - hmp −x)/(g¯ CaT − hmp −x)g¯ CaT −g¯ CaT - slope ))μScm−2

*x* represents radial distance from the soma. The evidence for non‐uniformity of passive properties (*R*_m_ and *R*_a_) comes from experimentally derived fitting procedures that have demonstrated the need for such non‐uniformity to match experimental outcomes (Stuart & Spruston, 1998; Poirazi *et al*. 2003a,*b*; Golding *et al*. 2005; Narayanan & Johnston, 2007) and from prior modelling studies that have employed non‐uniform passive properties (Poirazi *et al*. 2003a,*b*; Narayanan & Johnston, 2007; Narayanan *et al*. 2010; Ashhad & Narayanan, 2013; Rathour & Narayanan, 2014; Das & Narayanan, 2015; Sinha & Narayanan, 2015; Ashhad & Narayanan, 2016). The evidence for uniform distributions for NaF (Magee & Johnston, 1995; Bittner *et al*. 2012) and KDR (Hoffman *et al*. 1997), and for gradients in KA (Hoffman *et al*. 1997), HCN (Magee, 1998) and CaT (Magee & Johnston, 1995) channels, follows somatodendritic cell‐attached recordings from CA1 pyramidal neurons.

**Table 2:** Model parameters and their base values for the model

No.	Parameter (unit)	Symbol	Base value
*R*_a_ distribution			
1	Minimum value (Ω‧cm)	*R*_a_‐soma	120
2	Maximum value (Ω‧cm)	*R*_a_‐end	70
3	Half‐maximal point of sigmoid (μm)	*R*_a_‐hmp	300
4	Slope of sigmoid (μm)	*R*_a_‐slope	50
*R*_m_ distribution			
5	Minimum value (kΩ cm^–2^)	*R*_m_‐soma	125
6	Maximum value (kΩ cm^–2^)	*R*_m_‐end	850
7	Half‐maximal point of sigmoid (μm)	*R*_m_‐hmp	300
8	Slope of sigmoid (μm)	*R*_m_‐slope	50
Properties of spiking conductances			
9	Maximal conductance of fast sodium channel (mS cm^–2^)	${\bar{g}}_{\mathrm{Na}}$	16
10	Maximal conductance of delayed rectifier potassium channel (mS cm^–2^)	${\bar{g}}_{\mathrm{KDR}}$	10
*h* channel properties			
11	Maximal somatic conductance (μS cm^–2^)	${\bar{g}}_{h}$	25
12	Fold increase	${\bar{g}}_{h}$‐fold	12
13	Half‐maximal point of sigmoid (μm)	${\bar{g}}_{h}$‐hmp	320
14	Slope of sigmoid (μm)	${\bar{g}}_{h}$‐slope	50
*T*‐type Ca^2+^ channel properties			
15	Maximal somatic conductance (μS cm^–2^)	${\bar{g}}_{\mathrm{CaT}}$	80
16	Fold increase	${\bar{g}}_{\mathrm{CaT}}$‐fold	30
17	Half‐maximal point of sigmoid (μS cm^–2^)	${\bar{g}}_{\mathrm{CaT}}$‐hmp	350
18	Slope of sigmoid (μm)	${\bar{g}}_{\mathrm{CaT}}$‐slope	50
*A*‐type K^+^ channel properties			
19	Maximal somatic conductance (mS cm^–2^)	${\bar{g}}_{\mathrm{KA}}$	3.1
20	Fold increase per 100 μm	${\bar{g}}_{\mathrm{KA}}$‐fold	8

For the specific formulations of each passive/active gradient, see Table 1.

NaF and KDR conductances were distributed uniformly in the soma and across the dendritic arbor with respective maximal conductances set at ${\bar{g}}_{\mathrm{Na}}$ = 16 mS cm^–2^ and ${\bar{g}}_{\mathrm{KDR}}$ = 10 mS cm^–2^ (Magee & Johnston, 1995; Hoffman *et al*. 1997). The ${\bar{g}}_{\mathrm{Na}}$ in the axonal initial segment was five‐fold higher compared to the somatic value. The rest of the axon was considered to be passive. Because the recovery of dendritic sodium channels from inactivation is slower (Colbert *et al*. 1997), an additional inactivation gating variable was included in the model for Na^+^ channels that expressed in the apical dendrites (Migliore *et al*. 1999). The three subthreshold channel conductances (CaT, HCN and KA) were distributed with increasing somato‐apical gradients (Fig. 1 *B* and Table 1), as dictated by corresponding electrophysiological findings (Magee & Johnston, 1995; Hoffman *et al*. 1997; Magee, 1998). When incorporating electrophysiological observations on differences between the activation parameters of the KA channels in CA1 pyramidal cells (Hoffman *et al*. 1997), two different KA conductance models were employed for proximal (≤100 μm radial distance from soma) and distal (>100 μm) apical dendritic compartments (Migliore *et al*. 1999). The half‐maximal activation voltage for HCN channels was –82 mV for proximal apical compartments (radial distance ≤ 100 μm), linearly the voltage varied from –82 mV to –90 mV for compartments between 100 and 300 μm, and the voltage was set at –90 mV for compartments with distances larger than 300 μm (Magee, 1998). All active and passive properties of basal dendritic compartments were set to their respective somatic values.

All somatodendritic active and passive parameters and their gradients were tuned to match distance‐dependent electrophysiological measurements (Fig. 1 *C*–*G*) of back‐propagating action potentials (bAP), input resistance (*R*_in_), resonance frequency (*f*_R_) and total inductive phase (Φ_L_) from CA1 pyramidal neurons (Spruston *et al*. 1995; Hoffman *et al*. 1997; Narayanan & Johnston, 2007, 2008; Rathour & Narayanan, 2014).

### Intrinsic measurements

All intrinsic measurements (bAP, *R*_in_, *f*_R_ and Φ_L_) were computed using procedures described previously (Spruston *et al*. 1995; Hoffman *et al*. 1997; Narayanan & Johnston, 2007, 2008; Rathour & Narayanan, 2014). Briefly, to measure dendritic bAP, an action potential was initiated at the soma (2 nA current for 1 ms) and the bAP amplitude was measured at various locations along the somatoapical trunk (Fig. 1 *C*). *R*_in_ was measured by injecting subthreshold current pulses of amplitudes spanning –50 pA to +50 pA, in steps of 10 pA and recording the local voltage responses to these current pulses. The respective steady‐state voltage responses at a given location were plotted against the corresponding current amplitudes to obtain the *V*–*I* plot. The slope of a linear fit to this steady‐state *V*–*I* plot was taken as the *R*_in_ for that location, and the procedure was repeated for all locations along the somatoapical trunk (Fig. 1 *D*).

Impedance‐based measurements of the model were computed by injecting a chirp stimulus (Narayanan & Johnston, 2007, 2008; Rathour & Narayanan, 2012a, 2014): a sinusoidal current wave with constant amplitude (100 pA; peak‐to‐peak) with frequency linearly increasing from 0.1 to 15 Hz in 15 s (Fig. 1 *E*, top). The Fourier transform of the local voltage response (Fig. 1 *E*, bottom) was divided by the Fourier transform of the chirp stimulus (Fig. 1 *E*, top) to obtain the complex valued impedance *Z*(*f*), as a function of frequency *f*. The impedance amplitude profile |*Z*(*f*)| was computed as the magnitude of this impedance:

(1) Zf= Re Zf2+ Im Zf2

where  Re (Z(f)) and  Im (Z(f)) were the real and imaginary parts of the impedance |*Z*(*f*)|, respectively. The frequency at which |*Z*(*f*)| reached its maximum value was measured as the resonance frequency, *f*_R_, and was computed at each location along the somatoapical trunk (Fig. 1 *F*). The impedance phase profile ϕ(*f*) was computed as:

(2) ϕf=tan−1 Im Zf Re Zf

Φ_L_, defined as the area under the inductive part of ϕ(*f*), (Narayanan & Johnston, 2008), was computed for all locations along the somato‐apical trunk (Fig. 1 *G*) based on the local impedance phase profile:

(3) $$\Phi_{L}=\int_{\phi \left(f\right)>0}\phi \left(f\right)df$$

### Synaptic models and normalization of unitary potentials

Canonical synapses (default #synapses = 100) consisting of co‐localized NMDA and AMPA receptors were modelled using the GHK convention, with the default value of NMDAR:AMPAR ratio set at 1.5. The kinetics of AMPA and NMDA receptor currents were adopted from previous studies (Narayanan & Johnston, 2010; Ashhad & Narayanan, 2013; Anirudhan & Narayanan, 2015). The current through the NMDA receptor, as a function of voltage and time, was dependent on three ions: sodium, potassium and calcium. Consequently, as per the Goldman–Hodgkin–Katz convention:

(4) $$\begin{array}{ccc} I_{NMDA}\left(v,t\right) & \;=\; & I_{NMDA}^{Na}\left(v,t\right)+I_{NMDA}^{K}\left(v,t\right)\\ & & \;+I_{NMDA}^{Ca}\left(v,t\right) \end{array}$$

where

(5) $$\begin{array}{ccc} I_{NMDA}^{Na}\left(v,t\right) & \;=\; & {\bar{P}}_{NMDAR}P_{Na}s\left(t\right)MgB\left(v\right)\frac{vF^{2}}{RT}\\ & & \;\left(\frac{{\left[Na\right]}_{i}-{\left[Na\right]}_{o}\exp\left(-\frac{vF}{RT}\right)}{1-\exp\left(-\frac{vF}{RT}\right)}\right) \end{array}$$

(6) $$\begin{array}{ccc} I_{NMDA}^{K}\left(v,t\right) & \;=\; & {\bar{P}}_{NMDAR}P_{K}s\left(t\right)MgB\left(v\right)\frac{vF^{2}}{RT}\\ & & \;\left(\frac{{\left[K\right]}_{i}-{\left[K\right]}_{o}\exp\left(-\frac{vF}{RT}\right)}{1-\exp\left(-\frac{vF}{RT}\right)}\right) \end{array}$$

(7) $$\begin{array}{ccc} I_{NMDA}^{Ca}\left(v,t\right) & \;=\; & {\bar{P}}_{NMDAR}P_{Ca}s\left(t\right)MgB\left(v\right)\frac{4vF^{2}}{RT}\\ & & \;\left(\frac{{\left[Ca\right]}_{i}-{\left[Ca\right]}_{o}\exp\left(-\frac{2vF}{RT}\right)}{1-\exp\left(-\frac{2vF}{RT}\right)}\right) \end{array}$$

where ${\bar{P}}_{NMDAR}$ is the maximum permeability of the NMDA receptor. The relative permeability ratios were set at *P*_Ca_ =10.6, *P*_Na_ =1 and *P*_K_ =1. Default values of concentrations were (in mm): [*Na*]_i _= 18, [*Na*]_o _= 140, [*K*]_i _= 140, [*K*]_o _= 5, [*Ca*]_i _= 100 × 10^–6^ and [*Ca*]_o _= 2. *MgB(v)* governs the magnesium dependence of the NMDAR current, given as (Jahr & Stevens, 1990):

(8) $$MgB\left(v\right)={\left(1+\frac{{\left[Mg\right]}_{o}\exp\left(-0.062v\right)}{3.57}\right)}^{-1}$$

with the default value of [*Mg*]_o_ set at 2 mm. *s*(*t*) governs the kinetics of the NMDAR current, and is given as:

(9) $$s\left(t\right)=a\left(\exp\left(-\frac{t}{\tau_{d}}\right)-\exp\left(-\frac{t}{\tau_{r}}\right)\right)$$

where *a* is a normalization constant, making sure that 0 ≤ *s*(*t*) ≤ 1, τ_d_ is the decay time constant, τ_r_ is rise time, with τ_r_ =5 ms, and default τ_d_ =50 ms (Narayanan & Johnston, 2010; Ashhad & Narayanan, 2013).

Current through the AMPA receptor was modelled as the sum of currents carried by sodium and potassium ions:

(10) $$I_{AMPA}\left(v,t\right)=I_{AMPA}^{Na}\left(v,t\right)+I_{AMPA}^{K}\left(v,t\right)$$

where

(11) $$\begin{array}{ccc} I_{AMPA}^{Na}\left(v,t\right) & \;=\; & {\bar{P}}_{AMPAR}P_{Na}s\left(t\right)\frac{vF^{2}}{RT}\\ & & \;\left(\frac{{\left[Na\right]}_{i}-{\left[Na\right]}_{o}\exp\left(-\frac{vF}{RT}\right)}{1-\exp\left(-\frac{vF}{RT}\right)}\right) \end{array}$$

(12) $$\begin{array}{ccc} I_{AMPA}^{K}\left(v,t\right) & \;=\; & {\bar{P}}_{AMPAR}P_{K}s\left(t\right)\frac{vF^{2}}{RT}\\ & & \;\left(\frac{{\left[K\right]}_{i}-{\left[K\right]}_{o}\exp\left(-\frac{vF}{RT}\right)}{1-\exp\left(-\frac{vF}{RT}\right)}\right) \end{array}$$

where ${\bar{P}}_{\mathrm{AMPAR}}$ is the maximum permeability of the AMPA receptor. The relative permeability ratios *P*_Na_, *P* and *P*_K_ were equal and set to 1. *s*(*t*) was the same as that for the NMDA receptor but with τ_r _= 2 ms and τ_d _= 10 ms (Narayanan & Johnston, 2010). AMPAR permeabilities for synapses at any somato‐apical location (Fig. 1 *H*) were adjusted such that the unitary somatic response amplitude was ∼0.2 mV (Fig. 1 *I*), irrespective of synaptic location (Magee & Cook, 2000; Andrasfalvy & Magee, 2001). This ensured that attenuation along the dendritic cable did not play a critical role in determining the impact of synaptic localization profiles on tuning properties.

We also noted that the placement of these excitatory synapses on the dendrites, and not on explicitly modelled dendritic spines, was not relevant because the design criteria for synaptic strengths involved constraints on the unitary somatic response amplitude. Specifically, two scenarios are considered: one where the synapse was placed on the dendritic shaft and another where the same synapse was placed on a spine that was connected to this dendritic shaft. Assume that, to satisfy the constraint on somatic unitary EPSP being ∼0.2 mV, the local voltage response at the dendritic shaft was *V*_1_ mV when the synapse was placed on the shaft. Now, when the same synapse were placed on the spine, the receptor permeability for that synapse should be adjusted such that the somatic unitary EPSP is ∼0.2 mV. When this constraint is imposed, given the electrotonic characteristics of dendritic structures, this implies that the local voltage response at the dendritic shaft, even when the synapse was placed on the spine would be ∼*V*_1_ mV, thus abolishing the need for explicitly incorporating spines into dendritic structures.

### Place‐cell inputs and firing rate measurements

**Figure 2:**
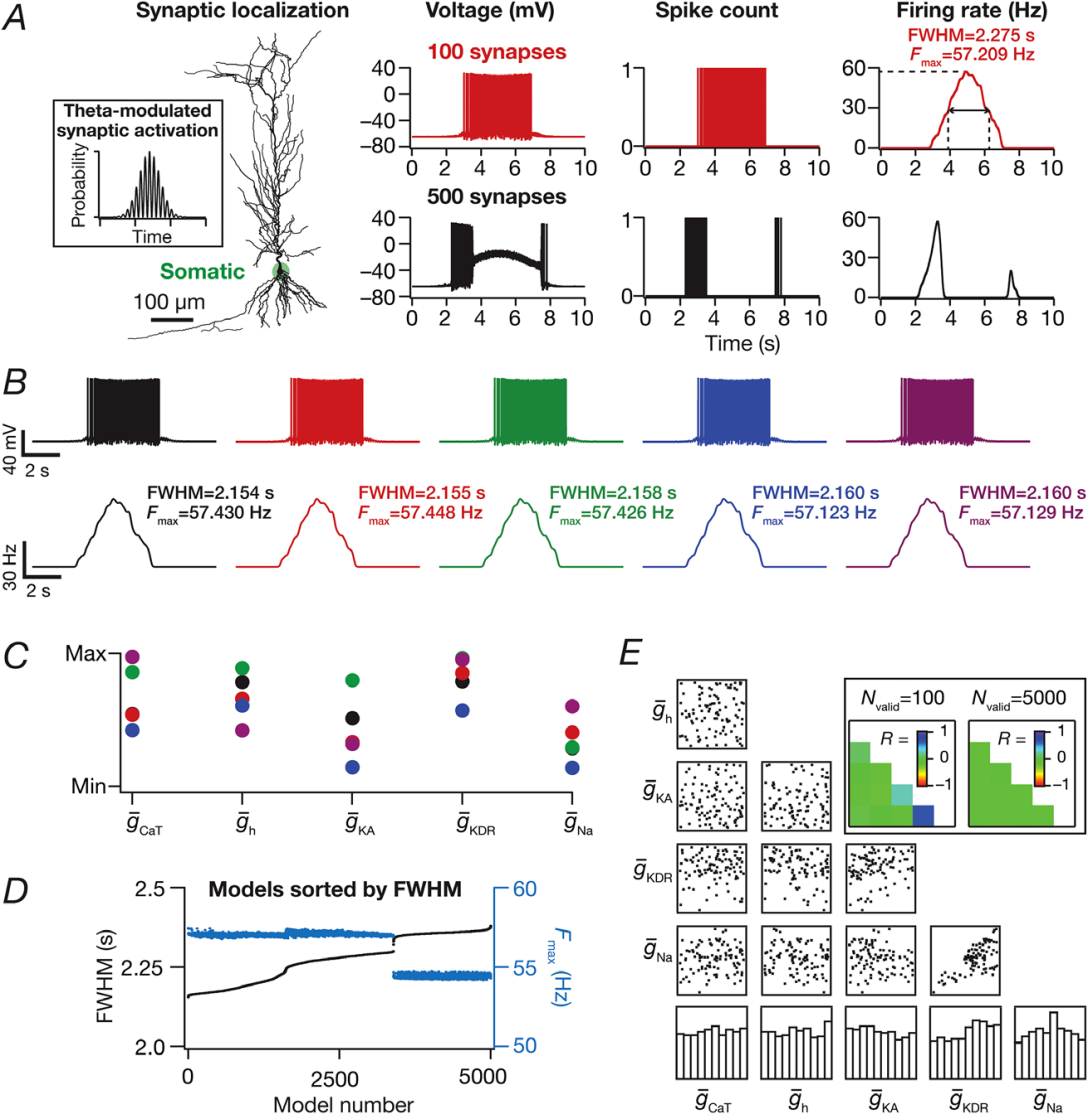
Place field synapses clustered on soma resulted in sharply‐tuned place cells with disparate combinations of voltage‐gated conductances *A*, left to right: morphological reconstruction of the model with synaptic localization highlighted; voltage traces obtained with 100 (top) and 500 (bottom) synapses; spike count plots (1 ms bin); firing rate profiles. *B*, five example voltage traces (top) and corresponding firing rate profiles (bottom) of valid models showing similar tuning. *C*, normalized parameter values of model cells shown in (*B*). *D*, FWHM and the corresponding *F*_max_ for all 5000 models, plotted in ascending sequence of their FWHM. *E*, scatter plot matrix and matrix representing corresponding Pearson's correlation coefficients (inset, left) for *N*_valid_ = 100 models. The lowest row of boxes depicts the distribution of each parameter for all 5000 models. The correlation coefficient matrix plotted for *N*_valid_ = 5000 models (inset, right) depicts a reduction in correlation coefficients with increase in *N*_valid_ (inset, left). *N*_syn_ = 100 for (*B*) to (*E*). [Color figure can be viewed at http://wileyonlinelibrary.com]

Place‐cell inputs were fed as probabilistic afferent activity impinging on the colocalized AMPAR‐NMDAR synapses described above. The frequency of place‐cell inputs to these synapses was modelled as an excitatory Gaussian modulated cosinusoidal current input driven through conductance‐based synapses, with the frequency of the sinusoid set at 8 Hz. This *conductance*‐based formulation was modified from (Geisler *et al*. 2010), where place‐cell inputs were modelled as a Gaussian‐modulated cosinusoidal *current*. This modification was essential because a current‐based input would not account for the driving‐force dependence of synaptic currents or the kinetics/voltage‐dependence of individual receptors (eqns (4), (5), (6), (7), (8), (9), (10), (11), (12)). Therefore, the total afferent current was modelled to arrive through multiple conductance‐based synapses whose presynaptic firing rates were probabilistically driven by a Gaussian‐modulated theta‐frequency cosinusoid representing the place field afferents. Each synapse in the neuron received inputs whose probability of occurrence at any given time point was defined by (Fig. 2 *A*, inset):

(13) $$\begin{array}{ccc} F_{pre}\left(t\right) & \;=\; & F_{pre}^{\max}\left(1+\cos\left(2\pi f_{0}\left(t-T\right)\right)\right)\\ & & \;\exp\left(-\frac{{\left(t-T\right)}^{2}}{2\sigma^{2}}\right) \end{array}$$

where *T* (5 s) defined the travel time between place field centres, *f*_0_ represented the cosine wave frequency (8 Hz), $F_{pre}^{\max}$ regulated the maximal input firing rate and σ defined the width of the Gaussian and controls the extent of the place field (1 s).

When such afferent activity from place fields (eqn (13)) impinged on synaptic receptors (eqns (4), (5), (6), (7), (8), (9), (10), (11), (12)) in models with different synaptic localization profiles (see below), current through these receptors drove post‐synaptic action potentials in a manner that was reflective of these place field inputs (Fig. 2 *A*). These spikes were converted to instantaneous firing rate profiles through convolution with a Gaussian kernel. Several measurements were derived from these firing rate profiles (Fig. 2 *A*): (i) the maximum firing rate of place cell (*F*_max_); (ii) the full‐width at half‐maximum (FWHM) of the profile, defined as the temporal distance between the two half‐maximal points (on either side of the centre) of the profile; and (iii) area under the curve (AUC) of the firing rate profile. These measurements were employed when comparing different place cell firing profiles, and to define sharpness of the tuning profile. Specifically, a low value of FWHM and a high value of maximal firing rate was considered to be indicative of a sharply tuned place cell response.

### Inhibitory synapses and their inputs

When incorporated, inhibitory inputs (*N*_syn _= 25) to model neurons were through synaptic GABA_A_ receptor (GABA_A_R) currents, modelled as a chloride current within the GHK formulation (Mishra & Narayanan, 2015):

(14) $$\begin{array}{ccc} I_{GABAA}^{Cl}\left(v,t\right) & = & {\bar{P}}_{GABAA}s\left(t\right)\frac{vF^{2}}{RT}\\ & & \left(\frac{{\left[Cl\right]}_{i}-{\left[Cl\right]}_{o}\exp\left(\frac{vF}{RT}\right)}{1-\exp\left(\frac{vF}{RT}\right)}\right) \end{array}$$

where ${\bar{P}}_{GABAAR}$ was the maximum permeability of the GABA receptor. These inhibitory synapses were randomly distributed perisomatically, within ∼50 μm of the somatic layer. *s*(*t*) was the same as that of the AMPA receptor with [*Cl*]_i_ = 5 mm and [*Cl*]_o_ = 98 mm, setting the reversal potential for GABA_A_ receptors at –80 mV. GABA_A_R permeabilities for synapses at any somatodendritic location were adjusted such that the unitary response amplitude was ∼ –1 mV, irrespective of synaptic location (Fig. 12 *A* and *B*). Similar to the excitatory afferent drive (eqn (13)), inhibitory inputs were modelled as probabilistic afferent activity impinging on the GABA_A_R synapses. The presynaptic frequency of these inputs was modelled as a Gaussian modulated cosinusoidal current input driven through conductance‐based synapses, with the frequency of the sinusoid set at 8 Hz. This cosinusoid was phase shifted by +60^o^ to account for theta phase shift between excitatory and inhibitory afferents (Buzsaki, 2002; Klausberger *et al*. 2003; Klausberger & Somogyi, 2008; Sinha & Narayanan, 2015):

(15) $$\begin{array}{ccc} F_{pre}\left(t\right) & \;=\; & F_{pre}^{\max}\left(1+\cos\left(2\pi f_{0}\left(t-T\right)+2/3\pi \right)\right)\\ & & \;\exp\left(-\frac{{\left(t-T\right)}^{2}}{2\sigma^{2}}\right) \end{array}$$

$F_{pre}^{\max}$ in this case was 0.6‐fold that of the excitatory input with the rest of the parameters identical to the excitatory input.

### Voltage ramps associated with place fields and theta‐frequency oscillations in place cell voltage traces

To assess the presence of characteristic ramps that place cells exhibit during place field traversal (Harvey *et al*. 2009), we subjected model voltage traces obtained during place field traversal (in response to synaptic inputs governed by eqn (13)) to a 0.75 s wide median filter. This ensured that the spikes were eliminated, thereby revealing the presence or absence of a voltage ramp (Fig. 8). The peak value of the voltage achieved by these ramps was employed as the maximum ramp voltage. Next, although the inputs to the afferent synapses to the neuron were theta‐modulated (eqn (13)), the somatic output voltage trace may or may not reflect this theta modulation as a result of dendritic filtering or the dominance of afterhyperpolarization conductances. To evaluate theta modulation in these voltage traces, we subjected model voltage traces obtained during place field traversal to a 50 ms wide median filter (a filter that replaced each voltage sample with the median of all neighbouring samples spanning 25 ms on either side). We performed spectral analysis on the resultant waveform and assessed theta modulation of this voltage response by finding the frequency at which the maximum power was observed (Fig. 8).

### Multiparametric multi‐objective stochastic search algorithm

A stochastic search algorithm spanning all critical parameters of the model has been employed as an effective method to (i) test the robustness of the system to parametric variability; (ii) determine whether there are parametric combinations where a specific set of measurement constraints are satisfied; (iii) find the relative sensitivities of different measurements to each parameter that forms the system; and (iv) explore pairwise and cross‐dependencies across different parameters towards achieving functional homeostasis. In such a global sensitivity analysis approach, each model parameter is assigned a range that spans multiple folds (on either side) of its value in a valid base model that is constrained by physiological measurements from the specific system under consideration. Then, in a given trial, each parameter is independently and randomly chosen through uniform sampling of its respective range to build a model and compute relevant measurements with this chosen set of parameters. Measurements from each of these models are then subjected to a test of validation to examine whether they are within their respective physiological bounds. Models that satisfy all the validation criteria are declared as valid models, and parametric combinations that resulted in these valid models can then be analysed to assess correlations and clustering in valid model parameters. Such parametric analyses, involving valid models that satisfy all functional requirements, provide insights about physiological constraints that need to be placed on model parameters for achieving the given set of functional constraints (Foster *et al*. 1993; Goldman *et al*. 2001; Prinz *et al*. 2004; Marder & Taylor, 2011; Rathour & Narayanan, 2012a, 2014; Anirudhan & Narayanan, 2015; Srikanth & Narayanan, 2015; Mukunda & Narayanan, 2017; Mittal & Narayanan, 2018).

Because such a stochastic search spans a large space involving multiple physiologically relevant model parameters and validates the resulting stochastic model based on multiple physiological objectives, the percentage of valid models attained through such a procedure is typically low. This translates to a requirement for an exhaustive stochastic search to arrive at a statistically relevant number of valid models. Conceptually, there are two possibilities as outcomes of this search strategy:

1. The search strategy *does* yield models that satisfy all the physiological objectives. In this scenario, valid models constitute solutions to the multiparametric multi‐objective optimization problem, with the parametric combinations that yielded these valid models typically employed to study the expression of degeneracy or the emergence of correlations across valid‐model parameters or assess the role of individual channels and their interactions in regulating physiology (Foster *et al*. 1993; Goldman *et al*. 2001; Prinz *et al*. 2004; Taylor *et al*. 2009; Marder & Taylor, 2011; Rathour & Narayanan, 2012a, 2014; Anirudhan & Narayanan, 2015; Srikanth & Narayanan, 2015; Mukunda & Narayanan, 2017; Mittal & Narayanan, 2018).
2. The search strategy *does not* yield models that satisfy all the physiological objectives. Interpretation of such a scenario is not straightforward because the absence of any valid model does not necessarily imply infeasibility of such a model configuration towards achieving all physiological objectives. This is simply consequent to the observation that the stochastic search does not *completely* cover the entire *N*‐dimensional parametric space, thereby allowing for the possibility where valid solutions could exist within the unexamined regions of the parametric space.

Here, we employ such a multiparametric multi‐objective stochastic search (MPMOSS) algorithm to assess the sensitivity of the sharpness of place cell tuning to synaptic and channel localization profiles. We employ distinct searches with different synaptic and channel localization profiles to find and assess models with similarly sharp place field tuning and similar intraneuronal functional maps.

### Sensitivity of place‐cell tuning to synaptic localization profile

**Figure 3:**
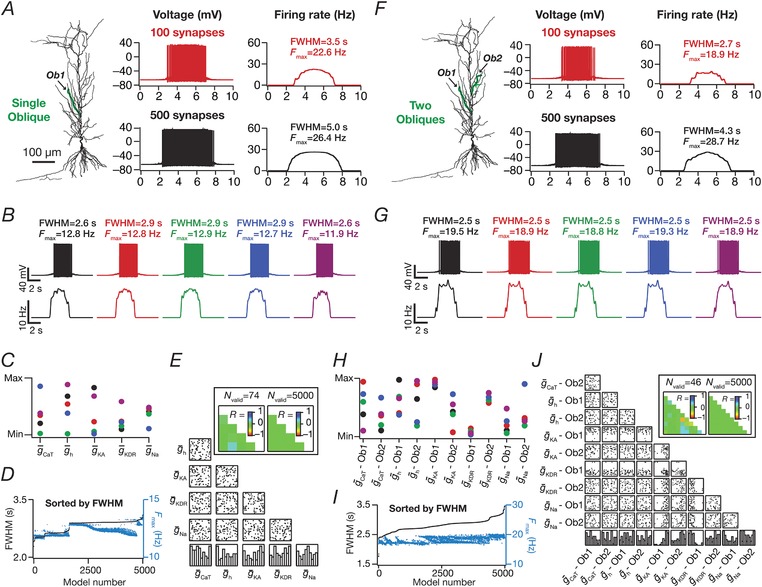
Place field synapses clustered on one or two obliques yielded weak place cell tuning with low firing rates with disparate combinations of local subthreshold channel conductances *A*, left to right: morphological reconstruction of the model with synaptic localization highlighted; voltage traces obtained with the different number of synapses; firing rate profiles. *N*_syn _= 100 (top), 500 (bottom). *B*, Five example voltage traces (top) and corresponding firing rate profiles (bottom) of cell models showing similar tuning profiles. *C*, normalized parameter values of model cells shown in (*B*). *D*, FWHM and corresponding *F*_max_ for all 5000 models, plotted in ascending sequence of their FWHM values. *E*, Scatter plot matrix and corresponding correlation coefficients (inset, left) for *N*_valid _= 74 similarly best‐tuned models. The lowest row of boxes depicts the distribution of each parameter for all 5000 models. *N*_syn _= 100 for (*B*) to (*E*). *A*–*E*, corresponding to synapses placed on a single apical oblique (*A*, *Ob1* branching from the trunk at ∼160 μm from the soma). *F*–*I*, same as (*A*) to (*E*) but with 50 synapses each clustered on two different obliques (*F*) (*Ob1* and *Ob2* branching from the trunk at ∼160 μm and ∼250 μm, respectively, from the soma) each. Scatter plots and correlation coefficients are for *N*_valid _= 46 similarly best‐tuned models. *E* and *I*, the correlation coefficient matrices plotted for *N*_valid _= 5000 models (inset, right) depicts a reduction in correlation coefficients with increase in *N*_valid_ (inset, left). [Color figure can be viewed at http://wileyonlinelibrary.com]

**Figure 4:**
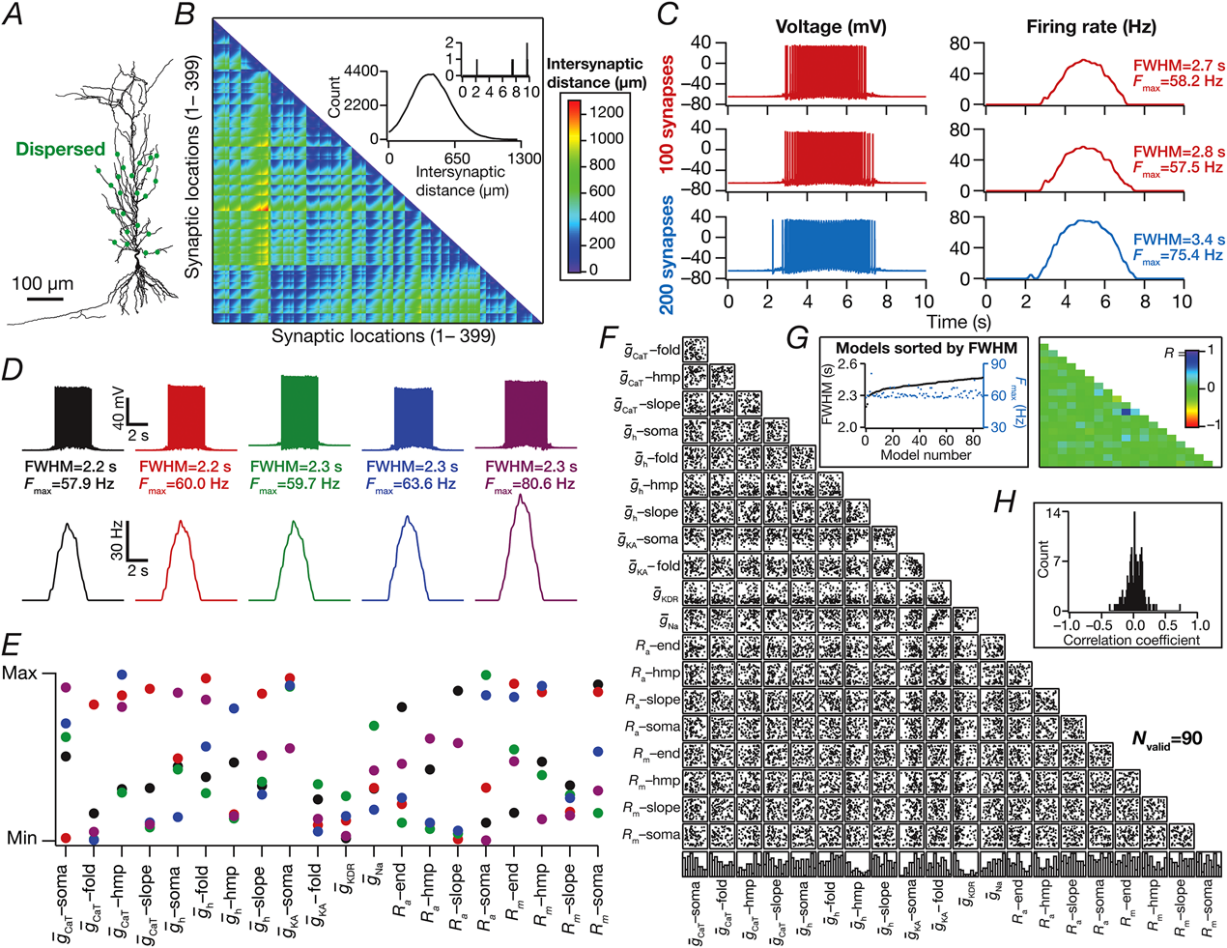
Place field synapses randomly dispersed across the dendritic tree yielded sharply‐tuned place cells with disparate combinations of active and passive parameters with weak pairwise correlations *A*, morphological reconstruction of the model with synapses distributed throughout the proximal 300 μm of the apical dendritic arbor (dots). *B*, lower triangular part of the intersynaptic distance matrix in models where synapses were randomly dispersed across the first 300 μm of the apical dendritic arbor. Inset: distribution of distances plotted in the matrix (top, zoomed to 0–10 μm, showing only four intersynaptic distances <10 μm). *C*, left to right: voltage traces obtained with 100 (top) and 200 (bottom) synapses; firing rate profiles. The two sets of traces correspond to two different randomized distributions of 100 synapses. *D*, five example voltage traces (top) and corresponding firing rate profiles (bottom) of valid models showing similar tuning profiles. *E*, normalized parameter values of model cells shown in (*D*). *F*, scatter plot matrix for *N*_valid _= 90 similarly best‐tuned models, obtained with spatially dispersed place field synapses (*N*_syn _= 100), depicting pairwise distributions between parameters. The lowest row of boxes depicts the distribution of each parameter for these valid models. *G*, left: FWHM and corresponding *F*_max_ for the 90 valid models, plotted in ascending sequence of their FWHM values. Right: Pearson correlation coefficient (*R*) matrix of the scatter plots in (*A*). *H*, distribution of correlation coefficients represented in (*G*). [Color figure can be viewed at http://wileyonlinelibrary.com]

We systematically assessed the impact of placing the same number of afferent synapses with identical impacts on somatic voltages (Fig. 1 *I*) with four distinct synaptic localization strategies: (i) all synapses were clustered at the soma (Fig. 2); (ii) all synapses were clustered within a single apical dendritic oblique (Fig. 3); (iii) synapses were clustered within two apical dendritic obliques (Fig. 3), with both the obliques receiving equal number of synapses; and (iv) synapses were randomly dispersed across the apical dendritic arbor (Fig. 4). In all cases, synapses received identical afferent inputs from a given place field location (eqn (13)), with their permeability adjusted in accordance with their somatodendritic location (Fig. 1 *H*) to normalize somatic impact of their activation (Fig. 1 *I*). These distinct synaptic localization profiles were systematically tested with different number of synapses (*N*_syn _= 10, 25, 50, 75, 100, 200 or 500) to analyse the sensitivity of place‐cell tuning to the number of synapses in each case (Fig. 7).

For each of these configurations of the synaptic localization, we employed independent stochastic search strategies on model parameters to assess the sensitivity of the combination of synaptic localization and channel conductances on place cell tuning sharpness. Each stochastic search strategy involved the generation of 5000 randomized models spanning different parametric spaces of channel conductances, with each channel conductance assigned a uniform search space of 0.5× to 2× of their respective baseline values (Table 2). As a part of the validation procedure of these stochastically generated models, we first removed all models that manifested a depolarization‐induced block in their voltage output (e.g. 500 synapses) (Fig. 2 *A*). This was achieved by plotting a histogram of membrane voltages, and rejecting models that showed a significant peak within the range of –45 mV to 50 mV. Following this, we sorted the models in two ways: first, an ascending order sort of their FWHM and, second, a descending order sequence of their maximal firing rate (*F*_max_). We selected models that were common to the first part of both the lists, such that they had a similarly small FWHM and a similarly high maximal firing rate, together implying similar sharp tuning of place fields. We took this relative approach to assessing tuning sharpness to ensure that our comparisons of the model remain focused on synaptic and channel localization profiles. Specifically, we assessed sharpness similarity in tuning profiles using relative comparisons of FWHM and *F*_max_ to circumvent heterogeneities in spatial extent of place‐cell populations, especially along the dorsoventral axis (Kjelstrup *et al*. 2008; Strange *et al*. 2014). Our experimental design involves the assessment of responses of the model cell to a Gaussian‐modulated cosinusoidal waveform (eqn (13)) with a *fixed* width. With the input distribution fixed, the design allowed us to focus specifically on the roles of the neuron's intrinsic properties and of synaptic localization on the output tuning profiles. The distribution of parameters in these selected valid models (with *similar* tuning profiles) and their pairwise correlations were then analysed to assess the robustness of the system to variability in channel properties and localization (Fig. 4).

### Balanced high‐conductance state

**Figure 5:**
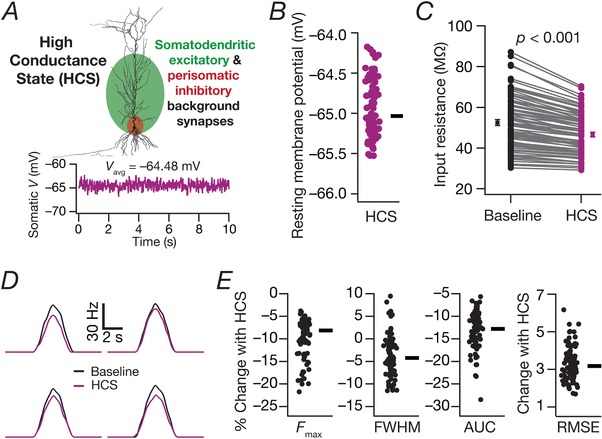
Effect of balanced high‐conductance state on sharply tuned place cells having distributed synaptic localization profile *A*, schematic representation of the methodology employed to introduce balanced high‐conductance state (HCS) through background synaptic activity impinging across locations spread through the neuronal arborization. Excitatory inputs impinged on all somatoapical compartments within a 300 μm distance from the soma, and inhibitory synapses made perisomatic contacts. Bottom: stochastic activation of these excitatory and inhibitory background synapses resulted in randomized fluctuations in the membrane voltage, with the excitation–inhibition balance maintained to yield an average baseline membrane potential of around –65 mV at the soma. *B*, beeswarm plot of average RMP for valid models from Fig. 4, when subjected to balanced HCS. The circles represent individual cells, and the rectangle depicts the median RMP across all cells. *C*, impact of balanced HCS on input resistance of the neuron without (Baseline) and with (HCS) the introduction of stochastic background synaptic activation (*P *< 0.001, paired Student's *t* test). Input resistance was computed from the steady‐state voltage deflection obtained with a single 500 pA hyperpolarizing current pulse lasting for 500 ms. Input resistance in the presence of HCS was computed as the average from 10 different trials with distinct randomizations of background synaptic activity (Mishra & Narayanan, 2015). *D*, firing rate profiles for four different model cells comparing neuronal responses in the absence (Baseline) or presence (HCS) of background synaptic activity. These responses were obtained when place field synapses (distinct from background synapses) in these models were activated with Gaussian‐modulated cosinusoidal firing. *E*, beeswarm plots (adjacent rectangles indicate corresponding median values) of changes in FWHM, *F*_max_, AUC and RMSE of the firing rate profiles obtained in the presence of background synaptic activity. Comparisons were made with the respective baseline model from Fig. 4. [Color figure can be viewed at http://wileyonlinelibrary.com]

For simulating background synaptic activity impinging on the neuron, we incorporated balanced excitation and inhibition to keep the average resting membrane potential (RMP) at ∼ –65 mV (Mishra & Narayanan, 2015). One excitatory synapse was placed at each compartment of the somato‐apical dendritic arbor within a 300 μm radial distance. Similarly, one inhibitory synapse was placed at each compartment within a radial distance of 50 μm perisomatically, including both apical and basal segments. For both the excitatory and inhibitory synaptic populations, independent random spike generators, each firing at an average rate of 4 Hz, was used for input stimulation of each synapse. All the synapses were modelled using an Ohmic formulation with the current through the synapse defined as:

(16) $$i_{syn}\left(t\right)=g_{syn}\left(t\right)\left(V-E_{R}\right)$$

where *g_syn_* (*t*) defined the time‐dependent evolution of each synapse after the onset of an afferent spike, and *E*_R_ defined the reversal potential for the synaptic receptors (*E*_R _= 0 mV for excitatory synapses and *E*_R _= –80 mV for inhibitory synapses). *g*_syn_ (*t*) was modelled using a double exponential synaptic formulation:

(17) gsynt=g¯exp−ttτdτd−exp−ttτrτr

where $\bar{g}$ defined the maximal conductance of each synapse set at 0.1 nS for excitatory synapses and 0.6 nS for inhibitory synapses. τ_r_ (= 2 ms) was the synaptic rise time constant and τ_d_ (= 10 ms) was the decay time constant for all the synapses. Upon stimulation with such randomized background activity, the mean somatic RMP was found to be –64.33 mV ± 0.74 mV (Fig. 5 *B*).

### Validation of models that manifested sharply tuned place field responses and matched physiological somatodendritic functional maps

When assessing the ability of disparate channel combinations to yield similarly tuned place‐cell responses and concomitantly match intrinsic physiological constraints on CA1 pyramidal neurons, we subjected models to a dual validation process. The first validation process, targeting place field encoding, picked model neurons that manifested sharply‐tuned place cell responses, specifically with high *F*_max_ and low FWHM. The second validation process focused on intrinsic functional maps across the somatoapical trunk (Rathour & Narayanan, 2014). Here, we calculated four intrinsic measurements for these models: bAP amplitude, input resistance (*R*_in_), resonance frequency (*f*_R_) and total inductive phase (Φ_L_), at soma, at ∼150 μm and at ∼300 μm along the somato‐apical dendritic trunk, giving a total of 12 constraining parameters. The neuronal models whose measurements fell within experimental boundaries noted in Table 3 were considered to be valid. Models that were validated for both sharp‐tuning and for intrinsic functional maps were then employed for further parametric analyses to assess the expression of channel degeneracy in achieving concomintant place‐cell encoding and excitability robustness.

**Table 3:** Bounds on 12 measurements for the model to be intrinsically valid

Measurement	Soma	∼150 μm	∼300 μm
bAP amplitude (mV)	90–115	40–70	5–45
Input resistance, *R*_in_ (MΩ)	40–100	30–60	10–50
Resonance frequency, *f*_R_ (Hz)	2–7	3–7	5–14
Total inductive phase, Φ_L_ (rad Hz)	0–0.3	0–1	0.025–2

Data from electrophysiological recordings reported in Spruston *et al*. (1995); Narayanan & Johnston (2007, 2008); Narayanan *et al*. (2010); and Malik *et al*. (2016).

### Sensitivity of place‐cell tuning to channel localization: virtual knockout simulations

Although channel localization gradients were fixed based on electrophysiological measurements (Fig. 1 *B* and Table 1), the dependence of place‐cell tuning to individual channel conductances was assessed through global sensitivity analysis involving the stochastic search strategy described above. In addition, to assess the specific role of individual channels on different place‐cell tuning measurements (FWHM, *F*_max_ and AUC of the firing rate profile), we performed virtual knockout simulations, where we virtually knocked out each of the different conductances in each valid model obtained through MPMOSS. This was implemented by independently setting the conductance value of each ion channel (performed separately for NMDA receptor permeability and for each of NaF, CaT, KA and HCN channel conductances) to 0, with all the other parameters intact from that specific valid model. Whereas the NaF channel knockout was specific only to apical dendritic compartments (the somatic, AIS and basal dendritic NaF channels were unchanged), all other knockouts spanned the entire somatodendritic arbor. The effect of each knockout was studied by comparing the measurements from the knockout model with respect to the corresponding valid model. This was repeated for all valid models for each knockout and the statistics of changes in place cell measurements were assessed across the valid model population to gain insights about the role of specific channels in determining place cell tuning profiles (Fig. 11).

### Dendritic spikes and bAP potentials

To assess the relative timing of spikes at dendritic and somatic locations, we recorded voltage traces at multiple locations along the apical trunk of each valid model obtained from all four MPMOSS algorithms (corresponding to the four synaptic localization strategies) described above. We computed the peak of the spike at several somatodendritic locations for each somatic action potential, detected at the upstroke of the somatic voltage crossing –20 mV. We calculated the differences between the timings of the peak at different dendritic voltages and the timing of the peak somatic voltage. Specifically, for five different apical trunk locations (100, 150, 200, 250 and 300 μm), we computed the following difference for each somatic spike:

(18) $$\begin{array}{ccc} \Delta T\left(sl,nv,tl,sp\right) & \;=\; & T_{tl}^{peak}\left(sl,nv,sp\right)\\ & & \;-T_{soma}^{peak}\left(sl,nv,sp\right) \end{array}$$

where Δ*T* represents the time difference, *sl* represents the synaptic localization strategy (somatic, one oblique, two obliques, dispersed), *nv* indexes the number of valid models (1, …, *N*_valid_) obtained with the specific synaptic localization strategy, *tl* represents the five different trunk locations and *sp* represents the current somatic spike index, and spans all the somatic spikes obtained for valid model number *nv*. $T_{tl}^{peak}$ and $T_{soma}^{peak}$, respectively, represented the time at which the spike reached its peak at location *tl* and at the soma. This difference was computed for all spikes in all valid models, and was binned into the appropriate synaptic localization profile *sl* and location *tl*.

A dendritic voltage peak occurring before the somatic voltage peak (negative Δ*T*) was considered to constitute a propagating dendritic spike at the recorded dendritic location, whereas a dendritic peak occurring after the somatic peak (positive Δ*T*) was representative of a bAP at that location. To quantify the fraction of dendritic spikes that resulted in a somatic action potential, we first plotted the histograms of Δ*T* for each location *tl* and each synaptic localization profile *sl* (Fig. 9 *E*). Because negative spike timings represented dendritic spikes occurring before somatic action potentials, we computed the fraction of the total area under the histogram curve that had negative Δ*T* and assigned that as the fraction of dendritic spikes preceding somatic action potentials. This value was computed for each *sl* and each *tl* to compare the impact of synaptic localization on dendritic spike generation (Fig. 9 *F*).

### Targeted synaptic plasticity in models receiving multiple place field afferents through randomized dispersed syanpses

We considered temporally sequential inputs from five different place fields, and randomly distributed 50 synapses for each of them (total 250 synapses) on the somato‐apical trunk of the CA1 pyramidal cell. With this synaptic configuration, we implemented the MPMOSS algorithm involving all the intrinsic parameters listed in Table 2 and an additional parameter (referred to as permfold, for fold change in permeability) to implement plasticity of synapses that are afferent from one of the five different place fields (arbitrarily chosen to be the second place field). Specifically, by default, the permeability values of all receptors were tuned to be peri‐threshold. The relative distance‐dependent gradients of receptor permeabilities (Fig. 1 *H*) were maintained when scaling these synaptic permeabilities to achieve default peri‐threshold excitability from all place fields. Then, as part of the MPMOSS strategy, the permeability values of the receptors associated with synapses receiving inputs from the second (of the five) place field were multiplied by a factor (*permfold*). The value of *permfold* was part of the stochastic search and was picked randomly from a uniform distribution spanning 1‐ to 5‐fold of the respective baseline permeability values. Specifically, the permeability values for synapses of the chosen place field could randomly vary from 1‐ to 5‐fold (the value of *permfold*) of their respective baseline perithreshold values, whereas the permeabilities for synapses from the other place fields remained at their baseline perithreshold values.

When implementing this MPMOSS strategy, we sampled this 21‐parameter search space to search for models and generated 2500 different models. Note that the span of these simulations were five times longer than the other simulations, as a result of the presence of five contiguous place field inputs impinging on the neuron. The aim here was to determine whether synaptic plasticity in one set of inputs was sufficient to elicit selectivity to a single place field input when multiple peri‐threshold place field inputs were impinging on the same cell through randomized dispersed synaptic localization (Lee *et al*. 2012; Bittner *et al*. 2015). Consequently, the validation criterion was to assess the selectivity of the neuron to the second place field (Fig. 13). Specifically, we declared a model to be valid if the peak firing rate of the neuron for inputs arriving from the second place field (in‐field firing rate) was at least 10 times the peak firing rate for inputs arriving from any of the other four place fields (out‐of‐field firing rate). Therefore, neurons that did not fire for any of the different place fields or neurons that fired across all place fields equally would be declared invalid. We chose a MPMOSS strategy that spanned both intrinsic properties and synaptic properties to explore degeneracy for achieving place field selectivity in scenarios where multiple place fields impinged on the same postsynaptic neuron.

### Computational details

All simulations were performed using the NEURON programming environment (Carnevale & Hines, 2006), at 34°C with the resting membrane potential set at –65 mV. The simulation step size was 25 μs. Data analyses and graph plotting were performed using custom‐written software in the IGOR Pro environment (WaveMetrics Inc., Portland, OR, USA).

## Results

As a first step in addressing questions on the impact of channel and synaptic localization profiles on sharpness of place‐cell tuning profiles, we employed a morphologically realistic conductance‐based model of a CA1 pyramidal neuron with channel distributions and physiological measurements (Fig. 1 and Table 1) that matched their electrophysiological counterparts (Magee & Johnston, 1995; Spruston *et al*. 1995; Hoffman *et al*. 1997; Magee, 1998; Narayanan & Johnston, 2007; Rathour & Narayanan, 2014). We scaled receptor conductances such that the unitary EPSP amplitude at the soma was of 0.2 mV amplitude (Fig. 1 *H* and *I*), irrespective of synaptic location across the dendritic arbor (Andrasfalvy & Magee, 2001). When systematically assessing the impact of synaptic localization profiles on place cell tuning, we placed synapses activated by the same place field at different locations across the somatodendritic arbor and computed sharpness of tuning in the neuronal firing rate for each of these localizations. To quantify the sharpness of the neuronal response, we employed two measurements: the FWHM and the *F*_max_ of the neuron's firing rate profile (Fig. 2 *A*).

### Models with synapses clustered at the soma elicited sharply‐tuned place fields with disparate combinations of channel conductances

As a first step, we placed several (*N*_syn _= 100 or 500) conductance‐based synapses, receiving afferent presynaptic activity as a Gaussian‐modulated theta waveform (eqn (13)) from the same place field (Geisler *et al*. 2010), on the soma (Fig. 2 *A*). We found that the base model was capable of eliciting sharp place‐cell‐like firing responses when *N*_syn_ was 100, although it entered depolarization‐induced block when *N*_syn_ was 500 (Fig. 2 *A*).

Was this sharp tuning that was obtained with somatic localization of synapses critically reliant on the specific conductance values set in the hand‐tuned base model? Were there explicit constraints on channel conductances to elicit sharply tuned place field responses with somatic localization of synapses? To explore this, we implemented a MPMOSS algorithm (Foster *et al*. 1993; Goldman *et al*. 2001; Marder & Taylor, 2011; Rathour & Narayanan, 2012a, 2014; Mukunda & Narayanan, 2017) on all the five active channel conductances (${\bar{g}}_{\mathrm{Na}}$ ,${\bar{g}}_{\mathrm{KDR}}$ ,${\bar{g}}_{\mathrm{KA}}$ ,${\bar{g}}_{h}$ and ${\bar{g}}_{\mathrm{CaT}}$ ) and their gradients (Table 2). We generated 5000 models with each of these maximal conductance values randomly picked from independent uniform distributions spanning 0.5‐ to 2‐fold of their respective base model values. We activated somatically localized synapses (*N*_syn _= 100) with stochastic place field inputs to each of these 5000 models and calculated the FWHM and *F*_max_ of their responses.

A representative set of five such model responses indicated similar sharp tuning across all these models (Fig. 2 *B*). However, the channel conductances that governed these models exhibited wide‐ranging variability (Fig. 2 *C*), implying that sharp place field tuning elicited by somatic localization of place field synapses was not critically reliant on the specific values of channel conductances. To further confirm this, we plotted the FWHM and *F*_max_ of all 5000 models and found minimal variability in these measurements, suggesting similar tuning (Fig. 2 *D*). Next, we picked 100 sharply‐tuned (low FWHM and high *F*_max_) models and investigated whether there were pairwise correlations between these model parameters. We found weak pairwise correlation coefficients (maximum *R *= 0.652; minimum *R *= –0.238; mean ± SEM = 0.05 ± 0.08) across all conductance values in these sharply‐tuned place cell models (Fig. 2 *E*). Because all models manifested similar tuning properties, we performed the correlation analysis for all 5000 models and, as expected (Rathour & Narayanan, 2014; Mukunda & Narayanan, 2017), the correlation values grew weaker with increase in the number of models (maximum *R *= 0.007, minimum *R *= –0.021 with *N*_valid _= 5000; mean ± SEM = –0.004 ± 0.002). Taken together, these results demonstrated that, with somatic localization of place field synapses, disparate channel combinations could yield similar tuning profiles with weak pairwise correlations between the underlying channel conductances.

### Spatially clustered inputs on one or two oblique dendrites did not confer sharpness in place cell tuning

Motivated by lines of evidence suggesting functional dendritic clustering of similar afferent inputs across different neurons (Takahashi *et al*. 2012; DeBello *et al*. 2014; Druckmann *et al*. 2014; Wilson *et al*. 2016), we placed synapses (*N*_syn _= 100 or 500) with identically stochastic (eqn (13)) activation profiles either on a single oblique or split equally across two distinct obliques. Irrespective of whether synapses were placed on one or two obliques, and irrespective of the number of total synapses, we found that the firing rate was low and the place field profile flat (Fig. 3 *A* and *F*), implying weak tuning of the place cell response. Given the critical role of oblique dendritic channel conductances in mediating dSpikes (Losonczy & Magee, 2006; Losonczy *et al*. 2008), could this conclusion be an artefact of the specific set of channel conductances in the obliques? To explore this possibility, we performed two different MPMOSS spanning all the conductances (${\bar{g}}_{\mathrm{CaT}}$ ,${\bar{g}}_{h}$ ,${\bar{g}}_{\mathrm{KA}}$ ${\bar{g}}_{\mathrm{KDR}}$ , and ${\bar{g}}_{\mathrm{Na}}$ ; *N*_syn _= 100) in one or two obliques, respectively, depending on whether synapses were localized onto one (five‐parameter MPMOSS) (Fig. 3 *B–E*) or two (10‐parameter MPMOSS) (Fig. 3 *G–J*) obliques.

A representative set of five such model responses indicated that the tuning was weak across these models, irrespective of whether synapses were localized on one (Fig. 3 *B*) or two (Fig. 3 *G*) obliques, although the corresponding channel conductances that governed these models manifested wide‐ranging variability (Fig. 3 *C* and *H*). This implied that the weak place field tuning elicited by synaptic localization on one or two obliques was not critically reliant on the specific value of oblique channel conductances (Fig. 3 *D* and *I*). We found weak pairwise correlation coefficients (one oblique: maximum *R *= 0.26; minimum *R *= –0.1; mean ± SEM = 0.009 ± 0.03, *N*_valid _= 74; two obliques: maximum *R *= 0.3; minimum *R *= –0.7; mean ± SEM = –0.02 ± 0.03, *N*_valid _= 46) across all conductance values in the similarly best tuned of these place cell models (Fig. 3 *E* and *J*). The correlation values grew weaker with increase in the number of models employed to compute the correlation coefficients (one oblique: maximum *R *= 0.007; minimum *R *= –0.02; mean ± SEM = 0.004 ± 0.002, *N*_valid _= 5000; two obliques: maximun *R *= 0.03; minimun *R *= –0.034; mean ± SEM = 0.0005 ± 0.002, *N*_valid _= 5000). Taken together, these results provided ample evidence for spatially clustered inputs that are confined to one or two dendritic branches being incapable of confering sharpness in place cell tuning.

### Dispersed synaptic inputs result in sharply tuned place cell responses

As a next step, we randomly distributed the same set of synapses (*N*_syn _= 100) throughout a large span of the apical region (radial distance from soma ≤300 μm) of the dendritic tree, with all of them receiving afferent place cell activity identically stochastic (eqn (13)) to earlier scenarios. There were 399 possible synaptic locations within this apical subregion with large intersynaptic distances (Fig. 4 *B*), which precluded the possibility of incidental clustering of synapses when they were randomly dispersed. We computed the somatic firing rate response and found place cell responses to be sharply tuned (Fig. 4 *C*; see also Fig. 2 *A*), irrespective of the specific randomization (within the 300 μm location) of dispersed synaptic localization (Fig. 4 *C*). When we increased the number of synapses to 200, we noted a reduction in spike height, especially at the centre of the place field as a consequence of high excitability and inadequate recovery of sodium channels from inactivation.

Was this conclusion on sharp tuning with dispersed synaptic inputs an artefact of the specific choice of channel conductances and their localization profiles in the base model? To address this, we performed an MPMOSS on 20 intrinsic parameters (Table 2) spanning 5000 models with randomly dispersed synaptic localization. We selected 90 of these models as best‐tuned valid models based on similarly low FWHM (<2.5 s) and high *F*_max_ (>55 Hz). We found that, despite the similarly sharp tuning profiles of five different cells picked from this population of 90 (Fig. 4 *D*), the underlying parametric distributions that defined these models covered the entire span of their respective ranges (Fig. 4 *E*). Finally, we assessed pairwise correlations of the parameters underlying the 90 valid models (Fig. 4 *F*–*H*) and found weak pairwise correlation across all assessed parameters (maximun *R *= 0.7; minimun *R *= –0.4, mean ± SEM = 0.01 ± 0.01, *N*_valid _= 90). Taken together, these results provide clear lines of evidence for multiple realizability of sharply tuned place field responses with dispersed synaptic localization profiles, with different randomized distributions of synapses and with disparate channel localization profiles.

### Sharpness of place cell tuning remains intact even in the presence of background synaptic activity in cells with dispersed synaptic localization

Do our conclusions hold true when the neuron is bombarded with background synaptic activity, as is the case *in vivo*? To test this, we took the valid models (Fig. 4) with dispersed synaptic localization (*N*_syn _= 100) obtained through MPMOSS. We then activated the place‐field synapses with the same Gaussian modulated theta input used so far but in the additional presence of background synaptic inputs yielding balanced high conductance state (Fig. 5 *A* and *B*). We compared the firing rate profiles of these cells with the respective control cells (i.e. without background synaptic activity from Fig. 4). As a direct consequence of the well‐established reduction in neuronal gain (Fig. 5 *C*) because of the introduction of high‐conductance states (Chance *et al*. 2002; Destexhe *et al*. 2003; Mishra & Narayanan, 2015), we observed a reduction in firing rate of model neurons to place field inputs (Fig. 5 *D* and *E*). However, the FWHM did not alter significantly and the profiles retained the sharp tuning (compare Fig. 3 *A* depicting a broadly‐tuned profile with Fig. 5 *D* with a sharply‐tuned profile) even in the presence of high‐conductance state. In summary, although the presence of high‐conductance states introduced an overall reduction in gain, the sharpness of tuning profiles was retained even in the presence of high‐conductance states.

### Ion channel degeneracy in the concomitant emergence of sharp place field tuning and intraneuronal functional maps

Our analyses thus far have been limited to the emergence of similarly tuned place field firing with various synaptic localization profiles. These analyses demonstrate that various configurations of randomly dispersed synaptic localization profiles coupled with disparate combinations of channel localization could yield similar tuning profiles encoding place field locations (Fig. 4). Although this points to degeneracy in the emergence of similarly tuned place field profiles, these analyses do not address the question of whether degeneracy could be a framework that enables the concomintant emergence of encoding at the same time as retaining the signature physiological characteristics of CA1 pyramidal neurons. More specifically, it has been demonstrated that several somatodendritic functional maps defining intrinsic responses characteristics of CA1 pyramidal neurons could emerge with disparate combinations of channel localization profiles (Rathour & Narayanan, 2014). Could these functional maps emerge with disparate channel combinations while also retaining similar sharp tuning when synaptic inputs are dispersed across the somatodendritic arbor?

To test this, we generated a total of 8000 neuronal models (including the 5000 described in Fig. 4) through random sampling of parameters listed in Table 2, with synaptic localization profile dispersed across the somatoapical arbor. These models were then subjected to a two‐step validation process (see Methods), with the first targeting place field encoding (high *F*_max_ and low FWHM of firing profiles) and the second focused on intrinsic functional maps (measurements and validation criteria listed in Table 3) across the somatoapical trunk. We obtained 152 models that showed similarly sharp place field tuning and were valid with the functional map constraints as well. We used these 152 models to assess ion channel degeneracy in the concomitant emergence of similar place field encoding profiles and experimentally validated intrinsic functional characteristics.

**Figure 6:**
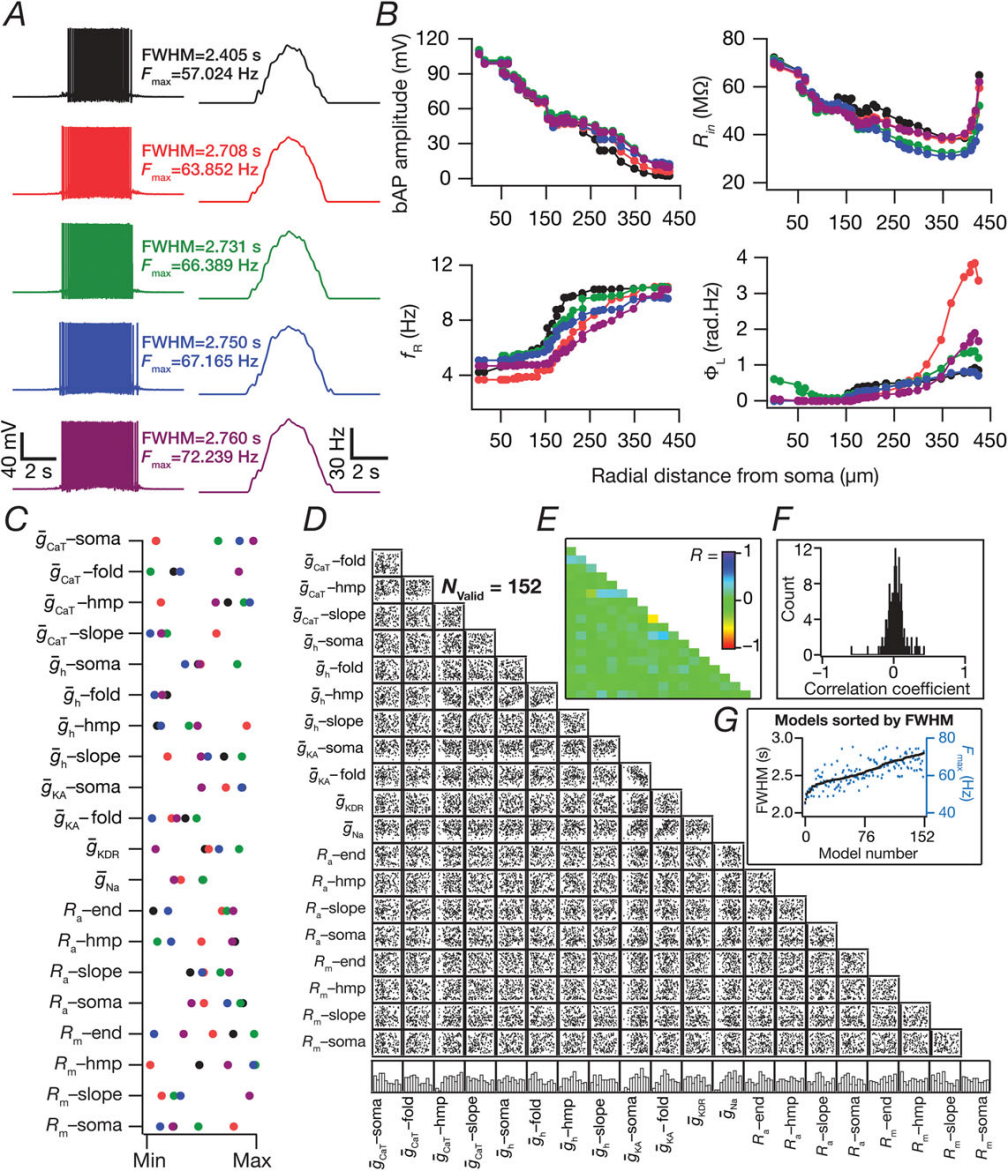
Ion channel degeneracy in the concomitant expression of sharp place‐cell encoding and robust intrinsic electrophysiological characteristics with dispersed synaptic localization profiles *A*, five example voltage traces (left) and corresponding firing rate profiles (right) of sharply‐tuned (low FWHM and high *F*_max_) and intrinsically‐valid (satisfied all 12 constraints in Table 3) neuronal models obtained after a stochastic search spanning 20 distinct model parameters (Table 2). The FWHM and *F*_max_ values are similar for all five models. *B*, complete functional maps along the somato‐apical trunk of the four validation measurements, namely, bAP amplitude, input resistance (*R*_in_), resonance frequency (*f*_R_) and total inductive phase (Φ_L_) for the five example cells shown in (*A*). The functional maps are similar for these five models. *C*, normalized parameter values of model cells shown in (*A*), demonstrating that disparate combinations of parameters yielding similar place‐cell selectivity (*A*) and intrinsic functional map characteristics (*B*). *D*–*E*, scatter plot matrix (*D*) and matrix representing corresponding correlation coefficients (*E*) for all 152 valid models (that satisfy both sets of sharp‐tuning and intrinsic functional map constraints), demonstrating weak pairwise correlations across parameters. The lowest row of boxes in (*D*) depicts the distribution of each parameter for all the valid models. *F*, the distribution of correlation coefficients depicted in (*E*) confirmed the absence of any strong pairwise correlations between the parameters. *G*, FWHM and corresponding *F*_max_ for the 152 valid models, sorted by FWHM. [Color figure can be viewed at http://wileyonlinelibrary.com]

We first picked five similarly tuned example model cells (Fig. 6 *A*) obtained after this two‐step validation process, which were also endowed with similar somatodendritic functional maps (Fig. 6 *B*). We investigated whether the parameters associated with these five example cells were clustered or were distributed across the span of their respective ranges (Table 2) and found them to be distributed (Fig. 6 *C*), constituting a line of evidence for ion channel degeneracy in the concomitant emergence of similar place field tuning and several intraneuronal functional maps. When we assessed the distribution of these parameters for all 152 models, we found the individual parametric histograms (Fig. 6 *D*, bottom) to be distributed across their respective ranges. In addition, similar to our earlier analyses involving only tuning profiles (Fig. 4), we also found weak pairwise correlations across parameters that defined these 152 models (Fig. 6 *D*–*F*; maximum *R *= 0.42; minimum *R *= –0.6; mean ± SEM = 0.02 ± 0.006). These results provide clear lines of evidence showing that sharp place cell tuning is attainable through dispersed synaptic localization and disparate channel localization profiles, concomitant to the robust emergence of distinct intraneuronal functional maps.

### Tuning sharpness and excitatory ramp in somatic voltage response were dependent on the number of synapses

**Figure 7:**
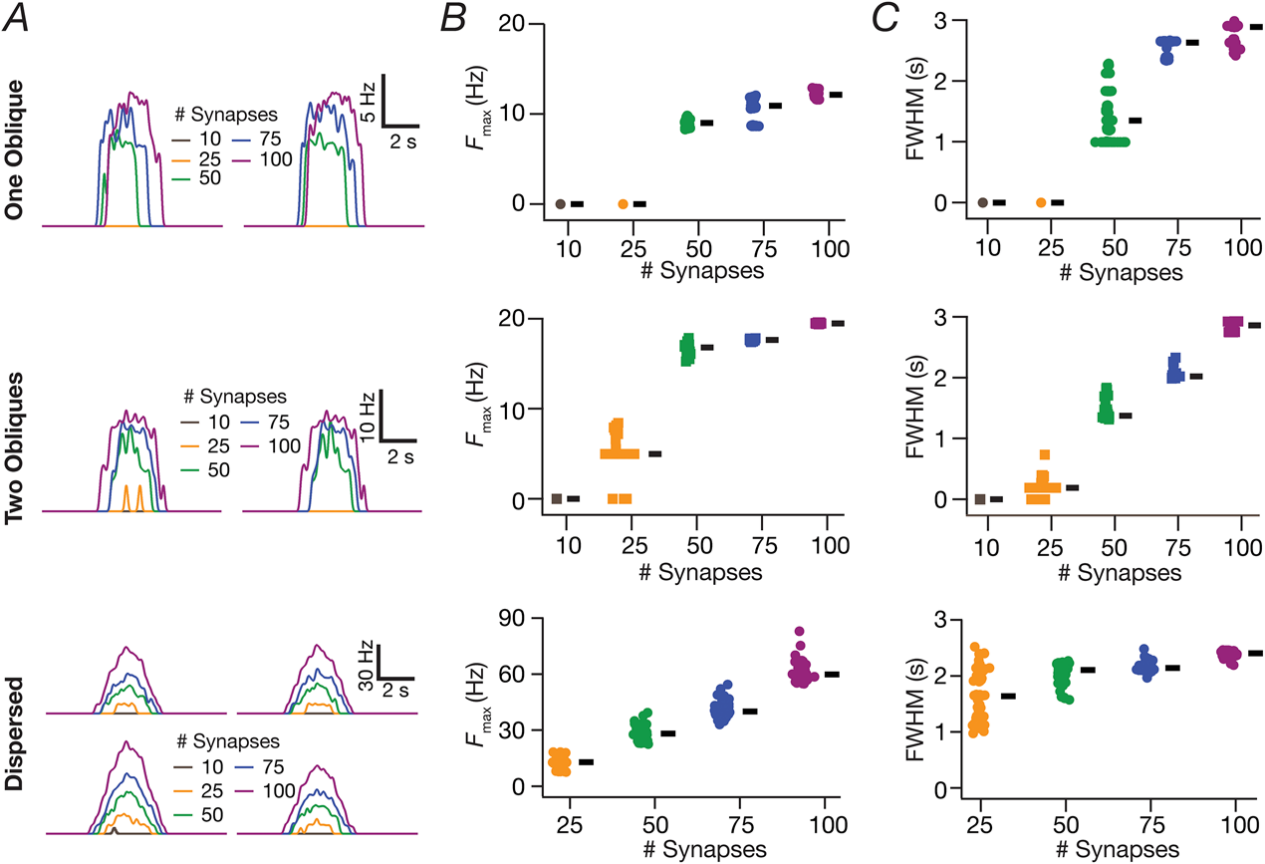
Effect of number of synapses on place cell tuning for clustered and dispersed synaptic localization *A*, instantaneous firing rate profiles for two example valid cell models for various number of synapses localized on a single oblique (top), two obliques (middle) or dispersed across the apical dendritic arbor (bottom). *B*, beeswarm plots of maximum firing rates (*F*_Max_) for all valid models with synapses localized on a single oblique (top), two obliques (middle) or dispersed across the apical dendritic arbor (bottom). *C*, beeswarm plots of FWHM for all valid models with synapses localized on a single oblique (top), two obliques (middle) or dispersed across the apical dendritic arbor (bottom). [Color figure can be viewed at http://wileyonlinelibrary.com]

How dependent were our conclusions on the number of synapses? Do these models exhibit an excitatory ramp in their somatic voltages, a characteristic feature in place cell recordings (Mehta *et al*. 2000; Harvey *et al*. 2009; Bittner *et al*. 2015)? Does the somatic voltage trace reflect the theta modulation from the synaptic drive that the neurons receive? Do answers to these questions depend on the specific synaptic localization profile? Would a reduction in number of synapses in the one‐ and two‐oblique localization cases enhance tuning sharpness? We employed valid models from the three distinct MPMOSS procedures (Figs 3 and 4) and found that the *F*_max_ and FWHM reduced with a reduction in the number of synapses, irrespective of synaptic localization profile (Fig. 7). Importantly, irrespective of the number of synapses associated with a place field, *F*_max_ was the largest and the FWHM was the least with the dispersed localization strategy compared to either synaptic clustering strategy.

**Figure 8:**
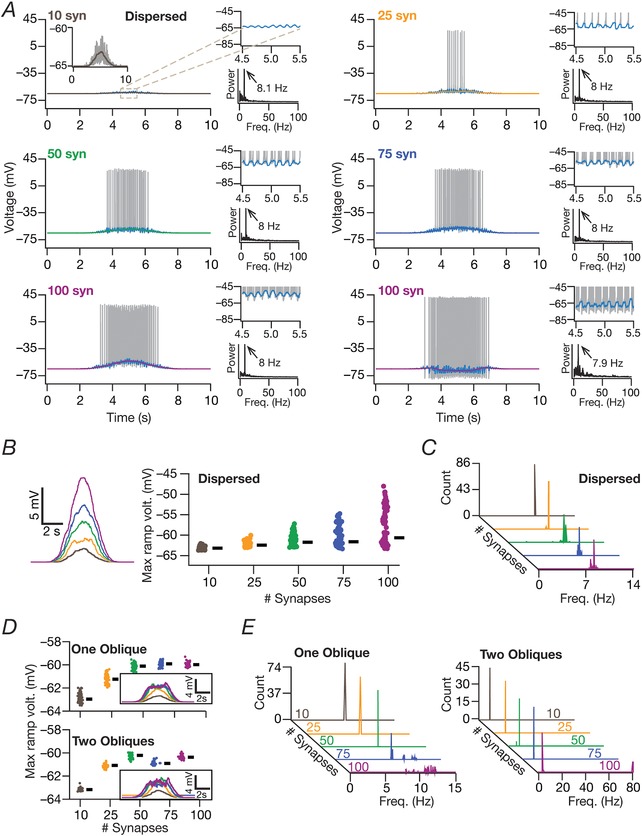
Models with place field randomly dispersed synapses exhibited excitatory voltage ramps, with the ramp amplitude and tuning sharpness dependent on the number of dispersed synapses *A*, for all subparts: left, excitatory ramp (filtered traces) overlaid on voltage traces of a valid cell model activated with different number of synapses. Right: top traces depict magnified views of a shorter time window at the place field centre, depicting the theta modulation of the ramp. The bottom plots depict the frequency spectrum of the filtered trace (over a 10 s time period) also depicting the frequency with maximum power (arrow). In a small subset of models (100 synapses, right), ramp‐like depolarization was suppressed by the dominant afterhyperpolarization dynamics. *B*, left: representative ramps for different numbers of synapses, same as ramp‐like traces shown in (*A*). Right, beeswarm plots of maximum ramp voltages for all valid models, plotted for different number of synapses (*N*_valid _= 87). Rectangles depict medians for the corresponding population. *C*, distributions of power frequency in theta‐smoothed ramps for all the valid models as a function of number of synapses. For (*A*) to (*C*), place field synapses were randomly dispersed. *D* and *E*, same as (*B*) to (*C*) but for models where place field synapses were clustered on one (*N*_valid _= 74) or two (*N*_valid _= 46) obliques. [Color figure can be viewed at http://wileyonlinelibrary.com]

Next, we smoothed somatic voltage traces of all valid models (from Figs 3 and 4), activated with different number of synapses, to eliminate spikes and determined whether they exhibited an excitatory ramp (Fig. 8). Although none of the models with any of the localization strategies elicited action potentials when they were activated with 10 synapses, they exhibited a subthreshold excitatory ramp of a few mV in amplitude (Fig. 8 *A*, *B* and *D*). As the number of activated synapses increased, the amplitude of the excitatory ramps expectedly increased, with much larger and sharper ramps achieved with the dispersed localization strategy than with the single‐ or double‐oblique clustering strategy (Fig. 8 *B* and *D*).

Finally, to assess theta modulation of somatic membrane potentials, we smoothed the somatic voltage traces enough to eliminate spikes but to retain theta oscillatory patterns. We performed Fourier analysis on these smoothened traces and found the peak frequency of these spectra (Fig. 8 *C* and *E*). We found strong ∼8 Hz (eqn (13)) power in most models with dispersed synaptic localization, irrespective of number of synapses employed (Fig. 8 *C*). However, when synapses were clustered within one or two obliques, we found several models where 8 Hz was not the dominant frequency in these smoothed voltage traces, especially when the number of synapses were higher (Fig. 8 *E*). These results implied that temporal precision within the theta range was preserved with dispersed synaptic localization but not when a large number of synapses were confined within one or two obliques. Taken together, these results demonstrated the remarkable effectiveness of dispersed synaptic localization for achieving sharp firing profile, sharp excitatory ramps and temporal precision in transfer of synaptic inputs, with such effectiveness invariant to the number of activated synapses.

### Dispersed synaptic localization was sufficient to elicit dendritic spikes

**Figure 9:**
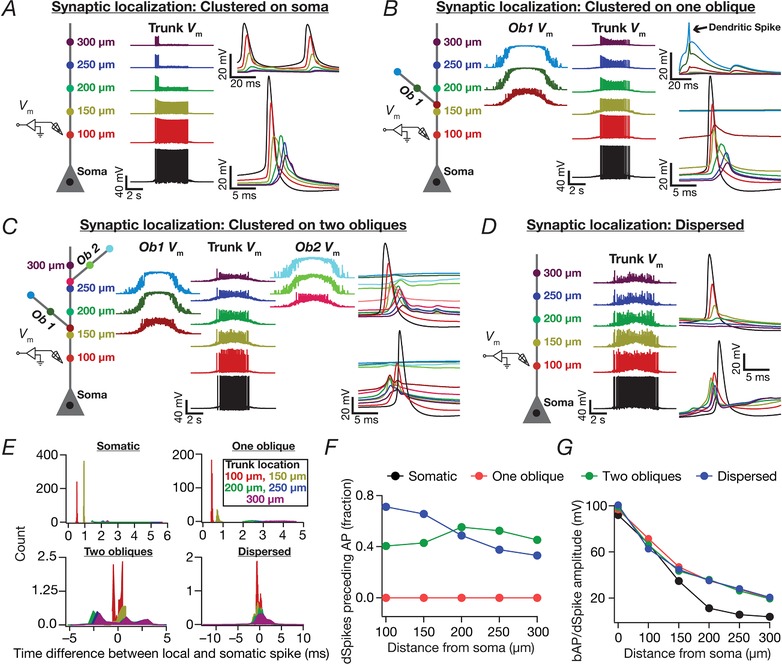
Spatially dispersed synapses yielded sharply‐tuned place cell models through dendritic spike initiation *A*–*D*, schematic of the neuronal somato‐apical arbor (left) showing various points where voltages were recorded (middle and right). *N*_syn _= 100 for all cases. *A*–*D*, differing in terms of synaptic localization profiles: (*A*) synapses clustered at soma. (*B*) synapses clustered on one oblique. Shown is a representative non‐propagating oblique dSpike (arrow). *C*, synapses clustered at two obliques (50 each). *D*, synapses dispersed throughout the somatoapical arbor. For (*C*) to (*D*), dendritic traces followed (top right) or preceded (bottom right) somatic traces for different somatic action potentials. *E*, distributions of between the timing of somatic action potential and dendritic bAP/dSpike along the somatoapical axis for the synaptic localization profiles analysed in (*A*) to (*D*). Note that negative values indicate the dendritic voltage peak preceding the somatic spike (dSpikes), whereas positive values indicate the somatic action potential to precede all other dendritic locations (bAPs). *F*, plots, derived from (*E*), depicting the fraction of somatic action potentials where the dendritically recorded spike preceded the somatic action potential. Note that the fraction of preceding dSpikes was zero for all locations when synapses were localized to either the soma or a single oblique. *G*, amplitudes of the peak voltages along the somatoapical axis corresponding to the different synaptic localization profiles. [Color figure can be viewed at http://wileyonlinelibrary.com]

What biophysical mechanisms were responsible for the sharp tuning of place cell responses in models with dispersed synaptic localization? Why did synaptic localization confined to one or two obliques not yield sharp tuning? Motivated by the evidence for the role of dSpikes in sharp tuning of place cells (Sheffield & Dombeck, 2015), we simultaneously recorded voltage traces at various points along the somatoapical arbor corresponding to each somatic action potential. We performed these recordings for the four different synaptic localization profiles and analysed these traces specifically for signatures of dSpikes (Fig. 9). Specifically, whereas a bAP would manifest as a dendritic voltage peak *following* the somatic action potential peak, a dSpike that potentially participated in action potential generation would express as a dendritic voltage peak that *precedes* the somatic action potential peak. When synapses were clustered at the soma (Fig. 2), as expected, all dendritic voltages recorded in conjunction with a somatic action potential were attenuating bAPs (Fig. 9 *A*) across all action potentials from all valid models (Fig. 9 *E* and *F*). Thus, with this physiologically unrealistic localization profile with no synapses in the dendrites, it was possible to achieve sharp spatial tuning without dSpikes.

**Figure 10:**
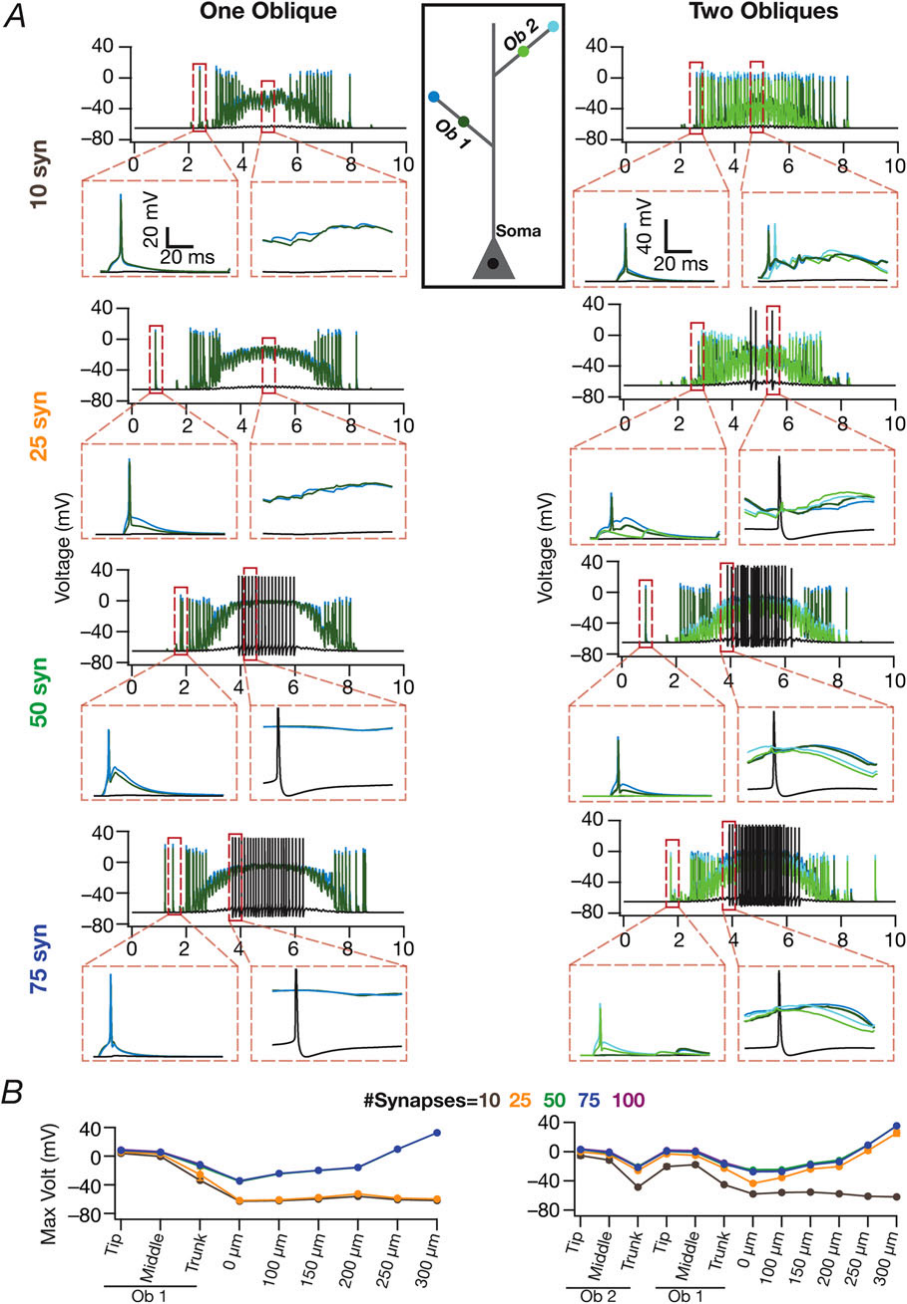
Effect of number of synapses on somatodendritic voltage traces for clustered synaptic localization *A*, left, Voltage traces recorded from the terminal end and at the centre point of the single oblique where all synapses were localized. Also shown are voltage traces concurrently recorded from the soma of the example valid cell model where synaptic localization was confined to a single oblique. The different subparts depict these traces for increasing number of synapses. Insets within dashed rectangles are magnified views of voltage traces from the corresponding highlighted segments. Right: same as the left, for models where synapses were equally distributed between two different oblique dendrites. The central inset (rectangle) shows the codes (same as Fig. 7 *B–C*) for the voltage traces shown on the left (only oblique 1) and the right (both obliques). *B*, peak recorded voltages for at different locations along the dendritic arbor, plotted (mean ± SEM) for models with different numbers of synapses. Shown are plots for models where synapses were confined either to one (left) and two (right) obliques (*N*_valid _– single oblique = 74, *N*_valid _– single oblique = 46). Note that the traces corresponding to 50, 75 and 100 synapses are overlapping on both graphs. Note also that, for lower numbers of synapses, the dendritic spikes initiated at the obliques do not travel to the cell body to initiate full‐blown action potentials. [Color figure can be viewed at http://wileyonlinelibrary.com]

We observed two distinct scenarios when synaptic localization was confined to one oblique, spanning all valid models (Fig. 3 *D*). First, during the initial part of the place field when the number of activated synapses was low because of the low probablity of afferent activation, there were dSpikes that were initiated in the oblique where the localization was confined to. However, consistent with *in vitro* evidence (Losonczy & Magee, 2006; Losonczy *et al*. 2008), these dSpikes did not result in a somatic action potential (Fig. 9 *B*, *E* and *F*). Second, with progressive increase in activation probability of synapses, the afferent excitation was sufficiently large to drive the oblique to depolarization‐induced block. This depolarization traversed through the dendritic arbor to elicit somatic action potentials, which backpropagated into the dendrites as bAPs (Fig. 9 *B*). Because somatic firing was elicited by the depolarization, and not by precisely timed dSpikes or synaptic potentials, the firing rate profile was weakly tuned (Figs 3, 9 *B*, *E* and *F* and 10). Although the overall scenario was similar to the one‐oblique case when synapses were localized on two obliques, there were cases where the dendritic voltage peak preceded the somatic action potential peak. These dSpikes were not distinctly observed in the two obliques where the synapses impinged and were observed on the trunk, which then propagated to the soma to elicit action potentials. However, the large depolarization‐induced block introduced in both obliques implied that the tuning was weak when synapses were localized to two obliques (Figs 3, 9 *C*, *E* and *F* and 10).

Could the depolarization‐induced block observed with single and double oblique localization profiles be because the synaptic drive was large? We repeated our voltage trace analyses with different number of synapses configured to these localization profiles. When the number of synapses was low, the oblique dendrites where synapses were placed showed dSpikes which did not propagate to the cell body (Fig. 10). With increase in number of synapses, the voltage profile within the oblique exhibited depolarization‐induced block, and this depolarization travelled to the soma to elicit weakly tuned spatial firing with low temporal precision (Fig. 8 *D* and *E*). Taken together, functional clustering on one or two oblique dendrites either generated localized dSpikes that did not result in a somatic action potential or resulted in a large depolarizing drive that yielded weakly tuned place field responses.

Strikingly, dSpikes preceding somatic action potentials were more prevalent in the scenario where synapses were dispersed across the dendritic arbor (Fig. 9 *D–F*). These results demonstrated that widespread depolarization coupled with large‐amplitude local EPSPs resulting from the high synaptic strengths (Fig. 1 *H* and *I*) was sufficient to elicit precisely timed dSpikes that together resulted in sharply tuned place field responses.

### Dendritic sodium channels, transient potassium channels and synaptic NMDARs were essential for sharp place cell responses with dispersed synaptic localization

**Figure 11:**
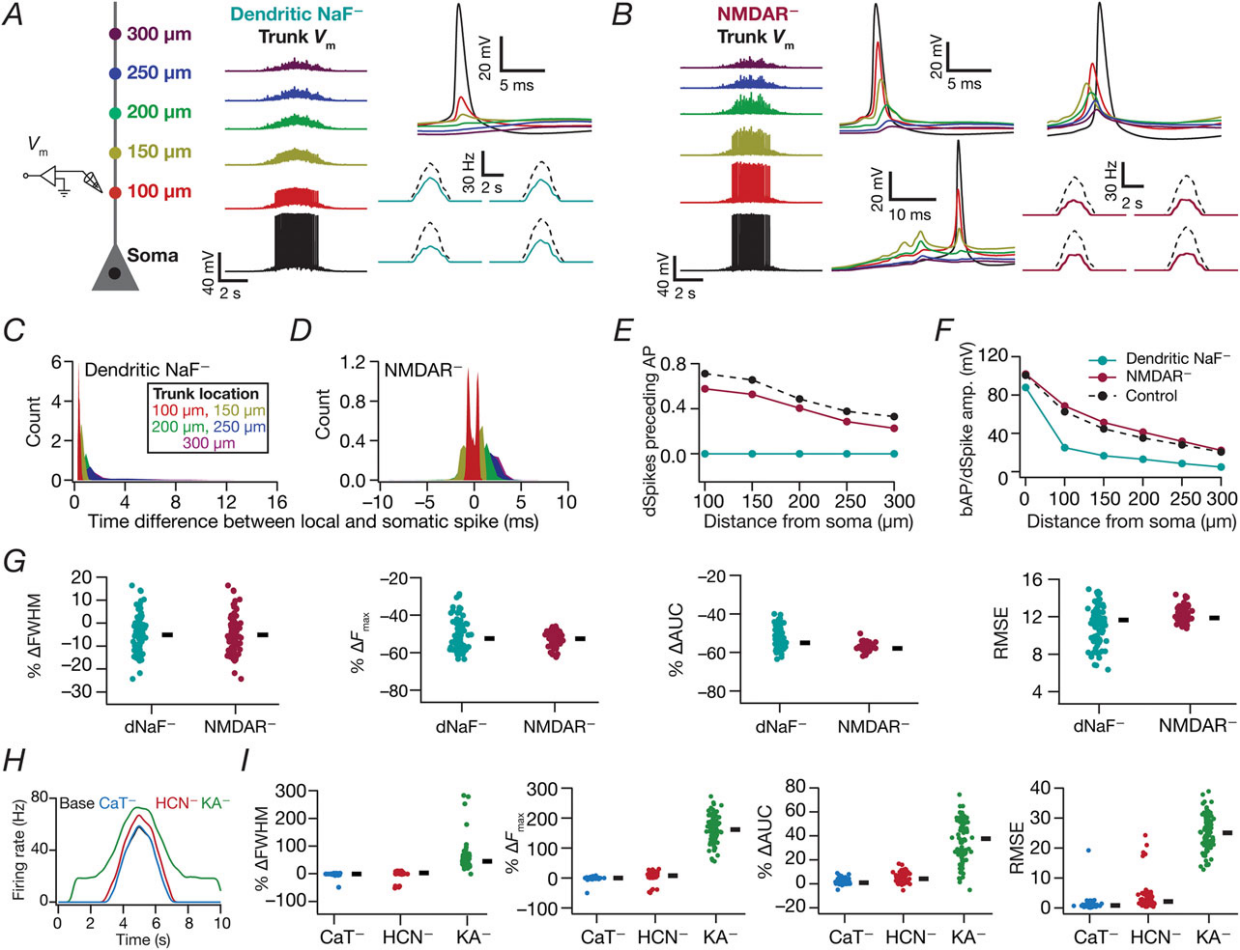
Place cell models with dispersed synaptic localization and disparate channel conductances lost sharpness of spatial tuning in the absence of dendritic sodium channels or NMDA receptors or transient potassium channels *A*, schematic of the neuronal somato‐apical arbor (left) showing various points where the voltages (middle) were recorded when apical dendritic sodium (dNaF) channels were knocked out. Firing rate profiles for four different model cells (right lower) comparing neuronal responses in the presence (dashed) and the absence (solid) of dNaF channels. *B*, voltage traces recorded from various points along the somato‐apical arbor (left: code as in *A*) when NMDA receptors were knocked out across all synapses. Example dendritic voltage traces whose peaks followed (middle top) or preceded (right top) somatic peaks for two different somatic action potentials are depicted. In some cases, dSpikes do not propagate to the soma to generate an action potential (middle bottom). Firing rate profiles for four different model cells (right lower) comparing neuronal responses in the presence (dashed) and the absence (solid) of NMDARs. *C*–*D*, distributions of between the timing of somatic action potential and dendritic bAP/dSpike along the somatoapical axis for the dNaF^–^ and NMDAR^–^ cases. *E*, plots derived from (*C*) to (*D*) depicting the fraction of somatic action potentials where the dendritically recorded spike preceded the somatic action potential. *F*, amplitudes of the peak voltages along the somatoapical axis corresponding to control models and for the dendritic NaF^–^ and NMDAR^–^ cases. *G*, beeswarm plots (rectangles are medians) of percentage changes in FWHM, *F*_max_, AUC and RMSE of the firing rate profiles obtained after virtual knockout of dNaF and NMDARs from each of the 90 valid models in Fig. 4. *H*, representative firing rate profile of a valid model and the profiles of the same model cell when different channels were knocked out. *I*, same as (*G*) for firing rate profiles obtained after virtual knockout of different channels from each of the 87/90 valid models in Fig. 4. [Color figure can be viewed at http://wileyonlinelibrary.com]

Are apical dendritic sodium channels and NMDARs essential for sharp place cell responses when synapses were dispersed? We employed the virtual knockout model (VKM) technique (Rathour & Narayanan, 2014; Mukunda & Narayanan, 2017) on each of the 87/90 valid models from the MPMOSS algorithm executed with dispersed synapses (Fig. 4). Specifically, we set the apical dendritic NaF conductance or NMDAR permeability in each of these valid models to zero and recorded model voltage and firing responses when identical (to the corresponding valid model) synaptic drive was afferent onto these models. We found that the absence of dendritic NaF channels or synaptic NMDARs resulted in a significant loss of tuning sharpness in these models (Fig. 11 *A–G*). As expected, there were no dSpikes when apical dendritic NaF channels were knocked out and bAPs did not spread significantly into the dendritic tree. Dendritic spikes were less prevalent in the absence of NMDARs (Fig. 11 *A–G*). These results suggest dendritic Na channels and NMDARs are essential ingredients for achieving sharp tuning with dispersed synaptic inputs. We also noted that, qualitatively, the reduction in firing rates observed with the absence of dendritic NaF channels or of NMDARs was similar to the outcomes that we had observed with lesser numbers of synapses (Fig. 7). This is to be expected because activation of dendritic NaF channels or NMDARs is critically reliant on number of functional synapses as a result of the voltage‐dependent activation profiles of NaF channels and NMDARs. This implies that a reduction in number of synapses translates to lesser activation of dendritic NaFs and NMDARs, resulting in lesser dendritic spikes when the number of synapses are reduced.

How sensitive was the sharpness of place cell tuning in valid models with dispersed synaptic localization to each subthreshold ion channel? We employed the VKM technique to measure place cell profiles in each of the 90 valid models by individually setting each subthreshold active conductances (${\bar{g}}_{\mathrm{CaT}}$, ${\bar{g}}_{h}$ , or ${\bar{g}}_{\mathrm{KA}}$ ) to zero. Although the impact of knocking out these different channels was differential across different models, knocking out the *A*‐type K^+^ channel yielded the maximum reduction in the sharpness of the place cell tuning (Fig. 11 *H*). This may be observed from the significant broadening of the firing rate profiles (example profile in Fig. 11 *H*; quantification of FWHM and other measurements in Fig. 11 *H* and *I*) after knockout of *A*‐type K^+^ channels. As expected, knocking out the HCN conductance resulted in a slight increase in excitability, whereas, on knocking out the CaT conductance, there was a slight decrease in excitability, although the impact of knocking out these channels was not as large as that for *A*‐type K^+^ channels (Fig. 11 *H* and *I*).

**Figure 12:**
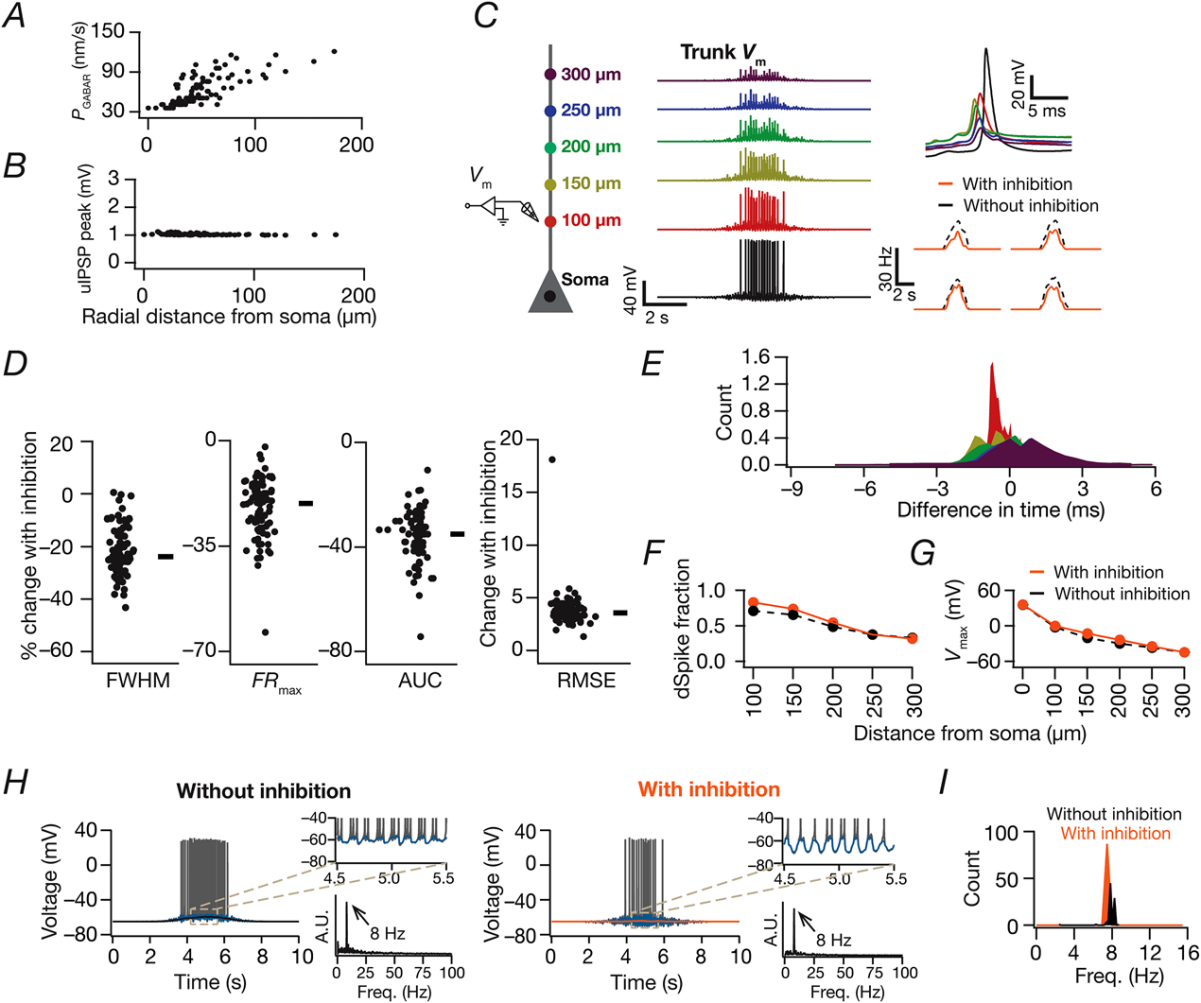
Effect of including theta‐modulated inhibitory synapses on place cell tuning for dispersed synaptic localization *A* and *B*, location‐dependent permeability values of GABAA receptor that normalized somatic unitary postsynaptic potential (uIPSP) amplitudes to around –1 mV as depicted in (*B*). *C*, schematic of the neuronal somato‐apical arbor (left) showing various points where the voltages were recorded (middle). Magnified, time aligned view of the voltage traces depicting an example dSpike (right upper) and firing rate profiles for four different model cells (right lower) comparing neuronal responses in the absence (dashed) and the presence (solid) of GABAARs. *D*, beeswarm plots (rectangles are medians) of percentage changes in FWHM, *F*_max_, AUC and RMSE of the firing rate profiles obtained after incorporating GABAAR synapses in each of the 87/90 valid models in Fig. 3. *E*, distributions of between the timing of somatic action potential and dendritic bAP/dSpike along the somatoapical axis. *F*, plot derived from (*E*) depicting the fraction of somatic action potentials where the dendritically recorded spike preceded the somatic action potential. *G*, amplitudes of the peak voltages along the somatoapical axis in models with or without inhibition. *H*, for models with and without inhibition; left: excitatory ramp (filtered traces) overlaid on voltage traces of an example valid cell model. Right: top traces depict magnified views of a shorter time window in the middle of the place field, depicting theta modulation in membrane voltage (filtered traces). The bottom plots depict the frequency spectrum of the filtered trace (over 10 s) also marking the frequency corresponding to the maximum power (arrows). *I*, distributions of power frequency in theta‐smoothed ramps for all valid models with or without inhibition. [Color figure can be viewed at http://wileyonlinelibrary.com]

Finally, to confirm whether our results were dependent on the presence of inhibitory synapses, we incorporated stochastically activated (eqn (15)) GABA_A_R synapses in the 87/90 valid models obtained with dispersed synaptic localization (Fig. 12). Although we observed an expected reduction in FWHM, *F*_max_, AUC and root mean squared error (RMSE) (Fig. 12 *D*), we noted the prevalence of dSpikes (Fig. 12 *C–G*), as well as precise temporal transfer (Fig. 12 *C–G*), in the presence of these inhibitory synapses.

### Targeted synaptic plasticity was sufficient to provide selective tuning to a single place field in neurons receiving randomly dispersed inputs from several place fields

If individual place fields projected to independent dendritic branches, clustered plasticity as a mechanism could enable enhancement of synapses only on one of these branches, thereby converting a silent cell (that receives input from several place fields) to a place cell that responds only to one specific location (Losonczy *et al*. 2008; Kleindienst *et al*. 2011; Makino & Malinow, 2011; Lee *et al*. 2012; Bittner *et al*. 2015; Bittner *et al*. 2017). However, if the synapses are dispersed as postulated here, then synapses corresponding to a given place field will be distributed all across the dendritic arbor, and would have to undergo simultaneous plasticity. It is feasible to envisage such a scenario because the induction of synaptic potentiation involves *temporal coincidence* of afferent synaptic activation and a postsynaptic depolarization (Magee & Johnston, 1997) without any specific requirement for spatial clustering. Nevertheless, could sharply tuned selectivity to one place field be achieved in neurons that receive afferent inputs from multiple place fields, each of which are randomly dispersed within the dendritic arbor, and are peri‐threshold in terms of their ability to elicit place cell responses? Would the overlaps in the spatial locations of synapses from different place fields, and their interactions with the underlying intrinsic somatodendritic properties preclude such selectivity even when synaptic plasticity is targeted only to synapses from one place field?

**Figure 13:**
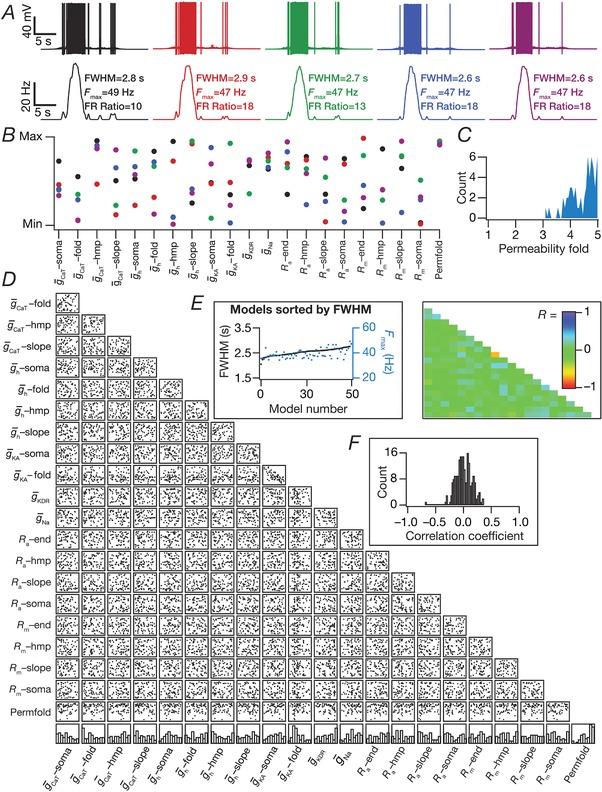
Targeted synaptic plasticity in afferents from a specific place field was sufficient to elicit place field selectivity in models that received randomly dispersed afferents from multiple place field locations *A*, five example voltage traces (top) and corresponding firing rate profiles (bottom) of valid models. Note that the firing rate of these model cells within the second place field was at least 10 times larger than their firing rates within any of the other place fields (FR Ratio: ratio between peak in‐field and peak out‐of‐field firing rates). *B*, normalized parameter values underlying models shown in (*A*) (same code). *C*, distribution of permeability folds (compared to base model permeability) for all the valid models (*N*_valid _= 60). *D*–*E*, scatter plot matrix (*D*) and matrix representing corresponding correlation coefficients (*E*) for the valid models. The lowest row of boxes in (*D*) depicts the distribution of each parameter for all valid models. FWHM and corresponding *F*_max_ for the 60 valid models obtained through MPMOSS, plotted in ascending sequence of their FWHM values (inset in *D*). *F*, distribution of correlation coefficients depicted in (*E*). [Color figure can be viewed at http://wileyonlinelibrary.com]

We independently and randomly dispersed synapses (50 synapses each) from five contiguous place field locations across the apical dendritic arbor. All of these place field inputs were peri‐threshold in the base model, making the model a silent cell. With this configuration set as the default base model, we executed an MPMOSS algorithm to generate 2500 models spanning 21 parameters, encompassing the 20 intrinsic parameters (Table 2) and an additional parameter (*permfold*) that governed potentiation of synapses associated with only one of the five (arbitrarily chosen to be the second place field) place field afferents. To validate these models, we accepted models that exhibited selective firing for the second place field and rejected those that either showed no firing or place‐non‐specific firing (Fig. 13 *A*). We noted that a significant number (1586 of 2500) of these models was rejected on this criteria of spatially‐selective firing, suggesting significant roles for complex interactions of intrinsic neuronal properties with synaptic potentiation and with spatially‐dispersed synapses from all place fields. This implies that models that exhibit spatial selectivity are non‐trivial demonstrations of how synaptic potentiation could convert a silent cell to a place cell when synapses from several place fields are randomly dispersed only under specific constraints on intrinsic properties. Among models that exhibited spatial selectivity, we then selected those with high *F*_max_ and low FWHM to obtain 60 valid models.

Are the afore‐mentioned constraints on intrinsic properties strong enough to preclude disparate channel combinations from eliciting similarly selective neuronal models? We chose five valid models that exhibited similar sharp tuning to the second place field (Fig. 13 *A*) and found significant variability in all model parameters, implying a lack of constraints on parameters towards eliciting place field selectivity and tuning sharpness (Fig. 13 *B* and *C*). The valid models also showed significant variability in the level of synaptic potentiation (3‐ to 5‐fold) required for meeting both validation criteria (Fig. 13 *C*), although several models with similar level of synaptic potentiation were rejected because of incompatible intrinsic properties. Finally, we observed weak pairwise correlation among the parameters (including with the *Permfold* parameter that defined the amount of synaptic potentiation), further emphasizing significant roles for complex interactions of intrinsic neuronal properties with synaptic plasticity in neurons receiving randomly dispersed inputs from several place fields. Taken together, these results suggest that variable levels of targeted synaptic potentiation, in conjunction with disparate sets of intrinsic properties, were sufficient to provide sharp and selective place field tuning in a cell that received randomly dispersed inputs from several place fields.

## Discussion

The prime conclusion of the present study is that randomly dispersed iso‐place field synaptic localization covering a wide span of dendritic arbor is sufficient to elicit dSpikes and sharply tuned place field selectivity in neuronal responses. We demonstrated this sufficiency with only a small proportion (*N*_syn _= 50–100) of synapses compared to those that typically impinge (15,000–30,000) on a pyramidal neuron (Bezaire & Soltesz, 2013), with each synapse physiologically constrained (Andrasfalvy & Magee, 2001) in terms of its unitary somatic EPSP. Employing MPMOSS, we demonstrated this sufficiency for different randomizations of dispersed synaptic localization and of disparate channel conductances. The dSpikes and the sharp tuning were critically dependent on dendritic sodium channels synaptic NMDA receptors and *A‐*type K^+^ channels, providing clear lines of quantitative evidence for a non‐linear mode of neuronal processing despite the dispersion in synaptic localization. Finally, we demonstrated that, despite randomly dispersed localization of synapses from several place field afferents, targeted synaptic potentiation was capable of eliciting sharp and selective tuning to one specific place field.

### Synaptic localization strategies and plasticity towards place cell formation

It is evident from our analyses that the prevalent dogma regarding the exclusiveness of linear *vs*. non‐linear modes of operation occuring, respectively, with dispersed *vs*. clustered synaptic localization ignored several features of hippocampal pyramidal neurons. First, the high‐density AMPARs in dendritic locations implies a large local dendritic voltage deflection corresponding to their afferent activation (Andrasfalvy & Magee, 2001). Second, the opening of slowly‐decaying, voltage‐dependent NMDARs following this large voltage deflection mediated by AMPARs manifests as a wider spread of a non‐linearly amplified depolarization (Schiller & Schiller, 2001) given that slower signals traverse longer and provide the substrate for better temporal summation (Rall, 1977). Third, given the extent of spatial influence of ion channels and given the interactions among depolarizations from multiple dispersed synapses (Rathour & Narayanan, 2012b), the combined large depolarizations emerging from the two receptor subtypes on each synapse were sufficient to cross the threshold for generating dSpikes (Fig. 9). Finally, converging depolarizations and propagating dSpikes from several concomitantly‐active dispersed synapses were sufficient to elicit temporally precise somatic action potentials yielding sharply tuned neuronal responses (Figs 4, 8 and 9).

With reference to clustered synapses, it is well established that the propagation of single oblique dSpikes, as resulting from spatiotemporally clustered synaptic activation, to yield somatic action potentials is weak, differential and state‐dependent (Golding & Spruston, 1998; Gasparini & Magee, 2006; Losonczy & Magee, 2006; Losonczy *et al*. 2008). The reasoning behind the link between clustered synaptic activation and a sharp tuning of neuronal responses is that spatial and temporal summation of multiple such oblique spikes manifests as sharply tuned neuronal firing responses. However, the case for temporal summation of dSpikes from *within* a single oblique is weakened by the presence of dendritic sodium channels for which the recovery from inactivation is very slow (Colbert *et al*. 1997). Such slow recovery from inactivation argues against fast repetitive generation of dSpikes in a single oblique, instead converting large afferent drives into a depolarization‐induced block (Figs 3, 9 and 10). Taken together, these translate into one of two alternatives when synapses (irrespective of their numbers) were clustered on one or two obliques: a non‐propagating oblique spike or a weakly‐tuned somatic response consequent to the depolarization traveling from the oblique (Figs 9 and 10).

In our analyses, we tested synaptic localization profiles confined to one or two oblique dendrites. However, the localization strategies form a continuum from the single oblique cluster to the randomized dispersion ends. Therefore, it is easy to extrapolate the possibility of attaining sharply tuned neural responses with the same number of synapses localized to multiple (not just one or two) obliques (Mel, 1993). This, in turn, highlights two distinct somatodendritic synaptic localization strategies towards achieving sharply tuned firing responses through the generation and propagation of dSpikes: random dispersion of synapses *vs*. specifically clustered localization limited to a few obliques for each perceptual feature.

Although either strategy would yield sharply tuned responses and it is possible that different neurons employ different strategies, we postulate that there are distinct advantages in choosing the dispersed localization strategy *especially* in the adult hippocampus where new place cells are formed in an activity‐dependent manner (Mehta *et al*. 1997; Bittner *et al*. 2015; Bittner *et al*. 2017). First, the rewiring and synapse‐formation requirements for targeting afferent synapses corresponding to a newly‐forming place field specifically onto a few obliques are more demanding than dispersing these synapses randomly across the dendritic arbor. Second, recent evidence indicates that synapses corresponding to different place fields impinge on silent cells (Lee *et al*. 2012; Bittner *et al*. 2015; Domnisoru & Tank, 2016; Bittner *et al*. 2017; Domnisoru & Tank, 2017). The presence of synapses from several place fields on a single neuron poses a resource allocation problem with reference to specific neuronal surface area assigned to each of these synaptic subsets. The randomized dispersed localization scenario provides a better solution to the allocation problem rather than assigning a multiple of the limited number of obliques to each of these different place fields, especially when the number of place fields to be allocated becomes large. This is possible because of the ability of the neuron to achieve robust tuning with different randomizations of dispersed synaptic profiles and with disparate channel conductances (Fig. 4).

Finally, from a plasticity perspective, the solution involving clusters on multiple obliques appears to offer a distinct advantage given the several demonstrations of clustered synaptic plasticity (Govindarajan *et al*. 2011; Makino & Malinow, 2011). However, plasticity involves *temporal coincidence* of afferent synaptic activation and a postsynaptic depolarization (Magee & Johnston, 1997), such as a widespread plateau potential that even invades the soma (Bittner *et al*. 2015; Bittner *et al*. 2017). Importantly, our results offer direct quantitative evidence for the ability of (variable levels of) targeted synaptic potentiation to achieve sharp selectivity to one place field even when multiple place field afferents impinge onto a neuron (with disparate channel properties) in a dispersed manner (Fig. 13). Taken together, our results offer an advantageous clear alternative to synaptic clustering, with significantly enhanced degrees of freedom, for achieving sharp feature selectivity through dSpike initiation with randomly dispersed iso‐feature synaptic afferents.

### Degeneracy in encoding systems: concomitant encoding and activity homeostasis

Degeneracy, the ability of disparate structural components to yield similar functional outcomes, is ubiquitous across biological process and has been linked to a robustness of physiology (Edelman & Gally, 2001; Leonardo, 2005; Whitacre, 2010; Whitacre & Bender, 2010; Drion *et al*. 2015; Rathour & Narayanan, 2017). However, most analyses of degeneracy are limited to the robust emergence of functional characteristics, with much less emphasis on whether and how degeneracy would assimilate into an encoding system where the ability to change is as important as the ability to retain robust homeostasis of intrinsic physiological characteristics (Rathour & Narayanan, 2017). Our analyses demonstrate that similar place field tuning could be obtained for an arbitrary place field location through targetted synaptic plasticity in neurons with disparate channel localization profiles and randomly dispersed place field synapses (Fig. 13). Importantly, in addition, we have also demonstrated that similar place field tuning profiles could concomitantly emerge along with the similar intrinsic functional maps, again with disparate channel localization and randomly dispersed place field synapses. These results provide clear lines of evidence showing that disparate channel and synaptic localization profiles could achieve similar place field tuning for distinct locations at the same time as maintaining intrinsic characteristics to specific physiological bounds.

Future experiments should focus on how degeneracy as a framework could effectively accommodate the need to change during encoding along with the need to remain constant in maintaining homeostasis (Rathour & Narayanan, 2017). With a specific focus on whether similarly tuned place fields with dispersed synapses and disparate channel localization could be obtained, experiments have shown that spines with different place tuning were interspersed within individual dendritic branches, and that even neighbouring spines could have place fields that were far apart in the environment (Domnisoru & Tank, 2017). Although this provides evidence for the possibility that synapses from a specific place field are dispersed, this does not assess the role of dendritic spikes or degeneracy in effectuating similar tuning profiles. One possible way to directly test this would be to introduce dispersed activation of afferent synapses through spatially and temporally precise patterned optogenetic activation (Packer *et al*. 2012; Grosenick *et al*. 2015; Shemesh *et al*. 2017) using *in vitro* electrophysiological experiments. Somatodendritic recordings of resultant firing profiles with disparate patterns of optogenetic activation coupled to temporal modulation of light power (Losonczy *et al*. 2010) as a Gaussian‐modulated cosinusoid (eqn (13)), in conjunction with the measurement of intraneuronal functional maps (Spruston *et al*. 1995; Narayanan & Johnston, 2007, 2008, 2012; Vaidya & Johnston, 2013) from the same neurons, would directly test whether distinct randomly dispersed synaptic profiles could yield similar tuning in distinct neurons with heterogeneous intrinsic properties. These experiments coupled to pharmacological agents targeting specific channels and receptors would also be effective in directly testing our predictions on the specific roles of different channels and receptors on tuning properties (Figs 11 and 12). Taken together, such experiments would further assess the ability of as a framework for the concomitant emergence of encoding and homeostasis (without cross‐interferences) in hippocampal pyramidal neurons.

### Limitations of the analyses and future directions

Although our model was severely constrained in terms of electrophysiological details from hippocampal pyramidal neurons (Figs 1 and 6), the model bears limitations in terms of other factors that could alter place‐cell tuning. First, because our focus is on localization and spatiotemporal summation of excitatory synaptic inputs, our analyses with inhibitory synaptic localization was not elaborate and we did not assess the role of dendritic inhibition in our model. However, dendritic inhibitory synapses play a critical role in the generation and propagation of dendritic spikes (Milstein *et al*. 2015; Grienberger *et al*. 2017). Second, absent in our model are several membrane and subcellular components, such as calcium stores on the endoplasmic reticulum, metabotropic receptors for different neurotrasmitters and neuromodulators, other channels such as the calcium‐activated potassium, G‐protein coupled and store‐operated calcium, and associated cross interactions (Nakamura *et al*. 1999; Narayanan *et al*. 2010; Wang *et al*. 2010; Ross, 2012; Ashhad & Narayanan, 2013; Ashhad *et al*. 2015; Grienberger *et al*. 2015; Prakriya & Lewis, 2015). Third, activation of synapses carrying place field information could lead to gliotransmision or release of retrograde messengers from the pyramidal neuron that could affect the dendritic and network integration processes (Regehr *et al*. 2009; Araque *et al*. 2014; Ashhad & Narayanan, 2016).

Finally, our model employs a deterministic framework for assessing channel interactions and their ability to elicit similar tuning and similar intrinsic properties. However, there is evidence that stochastic gating of dendritic ion channels could play a critical role in regulating the initiation of dendritic spikes (Cannon *et al*. 2010). Future studies should therefore experimentally determine the specific stochastic characteristics and associated dynamic evolution of native ion channels and receptors in CA1 pyramidal neurons, including somatodendritic heterogeneity in these signature characteristics. These experimentally‐determined stochastic ion channel models could then be incorporated to assess whether channel and synaptic noise of specific colors (with possible gradients in noise color across the somatodendritic axis of CA1 pyramidal neurons) alter the ability of neurons to elicit dendritic spikes in response to distinct afferent inputs. These limitations and missing components also make a critical case for their incorporation into future models with different synaptic (e.g. dendritic inhibitory and neuromodulatory synapses) localization profiles, including their stochastic short‐term synaptic dynamics (Pan & Zucker, 2009; Nadkarni *et al*. 2010; Nadkarni *et al*. 2012; Regehr, 2012; Mukunda & Narayanan, 2017). However, the several electrophysiological constraints that we had imposed on our model and the conclusions from our broad array of sensitivity analyses are fairly strong to allow the validity of our conclusions even when these other components are considered.

Although our conclusions are largely extendible to pyramidal neurons in other brain regions, the specifics of morphological characteristics, passive properties, ion channels and receptors expressed there should be rigorously assessed before formulating such an extrapolation. For example, several cortical pyramidal neurons exhibit characteristic up‐down states and the generation of dSpikes through the activation of very few synapses is sufficient to elicit a somatic action potential during up states (Palmer *et al*. 2014). However, hippocampal neurons do not exhibit up‐down states and the cell rests at hyperpolarized resting voltages before the animal enters the place field of the cell that is being recorded (Harvey *et al*. 2009; Lee *et al*. 2012; Bittner *et al*. 2015). Because the action potential threshold voltage is typically tens of millivolt above threshold, this configuration rules out the possibility of the activation of a few synapses resulting in well‐tuned somatic responses in hippocampal neurons. However, given the expression of up‐down states in cortical neurons, this should be considered possible in cortical pyramids during up states (Palmer *et al*. 2014). Future studies should explore the similarities and differences between feature selectivity in different pyramidal neurons across the sensory‐perceptual systems with specific focus on the characteristic channels and synaptic properites in different neuronal structures.

## Additional information

### Conflict of interest

The authors declare that they have no competing interests.

### Author contributions

RB and RN designed the experiments. RB performed the experiments and carried out the data analysis. RB and RN co‐wrote the paper. All authors have approved the final version of the manuscript submitted for publication.

### Funding

This work was supported by the Wellcome Trust‐DBT India Alliance (Senior fellowship to RN; IA/S/16/2/502727), the Department of Biotechnology (RN), the University Grants Commission (RB) and the Ministry of Human Resource Development (RN).

## References

[tjp13069-bib-0001] Andrasfalvy BK & Magee JC (2001). Distance‐dependent increase in AMPA receptor number in the dendrites of adult hippocampal CA1 pyramidal neurons. J Neurosci 21, 9151–9159. 11717348 10.1523/JNEUROSCI.21-23-09151.2001 PMC6763889

[tjp13069-bib-0002] Anirudhan A & Narayanan R (2015). Analogous synaptic plasticity profiles emerge from disparate channel combinations. J Neurosci 35, 4691–4705. 25788686 10.1523/JNEUROSCI.4223-14.2015 PMC6605137

[tjp13069-bib-0003] Araque A , Carmignoto G , Haydon PG , Oliet SH , Robitaille R & Volterra A (2014). Gliotransmitters travel in time and space. Neuron 81, 728–739. 24559669 10.1016/j.neuron.2014.02.007 PMC4107238

[tjp13069-bib-0004] Ascoli GA , Donohue DE & Halavi M (2007). NeuroMorpho.Org: a central resource for neuronal morphologies. J Neurosci 27, 9247–9251. 17728438 10.1523/JNEUROSCI.2055-07.2007 PMC6673130

[tjp13069-bib-0005] Ashhad S , Johnston D & Narayanan R (2015). Activation of InsP3 receptors is sufficient for inducing graded intrinsic plasticity in rat hippocampal pyramidal neurons. J Neurophysiol 113, 2002–2013. 25552640 10.1152/jn.00833.2014 PMC4416566

[tjp13069-bib-0006] Ashhad S & Narayanan R (2013). Quantitative interactions between the A‐type K^+^ current and inositol trisphosphate receptors regulate intraneuronal Ca^2+^ waves and synaptic plasticity. J Physiol 591, 1645–1669. 23283761 10.1113/jphysiol.2012.245688 PMC3624844

[tjp13069-bib-0007] Ashhad S & Narayanan R (2016). Active dendrites regulate the impact of gliotransmission on rat hippocampal pyramidal neurons. Proc Natl Acad Sci U S A 113, E3280–E3289. 27217559 10.1073/pnas.1522180113 PMC4988595

[tjp13069-bib-0008] Bezaire MJ & Soltesz I (2013). Quantitative assessment of CA1 local circuits: knowledge base for interneuron‐pyramidal cell connectivity. Hippocampus 23, 751–785. 23674373 10.1002/hipo.22141 PMC3775914

[tjp13069-bib-0009] Bittner KC , Andrasfalvy BK & Magee JC (2012). Ion channel gradients in the apical tuft region of CA1 pyramidal neurons. PloS ONE 7, e46652. 23056387 10.1371/journal.pone.0046652 PMC3463549

[tjp13069-bib-0010] Bittner KC , Grienberger C , Vaidya SP , Milstein AD , Macklin JJ , Suh J , Tonegawa S & Magee JC (2015). Conjunctive input processing drives feature selectivity in hippocampal CA1 neurons. Nat Neurosci 18, 1133–1142. 26167906 10.1038/nn.4062 PMC4888374

[tjp13069-bib-0011] Bittner KC , Milstein AD , Grienberger C , Romani S & Magee JC (2017). Behavioral time scale synaptic plasticity underlies CA1 place fields. Science 357, 1033–1036. 28883072 10.1126/science.aan3846 PMC7289271

[tjp13069-bib-0012] Buzsaki G (2002). Theta oscillations in the hippocampus. Neuron 33, 325–340. 11832222 10.1016/s0896-6273(02)00586-x

[tjp13069-bib-0013] Cannon RC , O'Donnell C & Nolan MF (2010). Stochastic ion channel gating in dendritic neurons: morphology dependence and probabilistic synaptic activation of dendritic spikes. PLoS Comput Biol 6, e1000886. 20711353 10.1371/journal.pcbi.1000886 PMC2920836

[tjp13069-bib-0014] Carnevale NT & Hines ML (2006). The Neuron Book. Cambridge University Press, Cambridge.

[tjp13069-bib-0015] Chance FS , Abbott LF & Reyes AD (2002). Gain modulation from background synaptic input. Neuron 35, 773–782. 12194875 10.1016/s0896-6273(02)00820-6

[tjp13069-bib-0016] Chen X , Leischner U , Rochefort NL , Nelken I & Konnerth A (2011). Functional mapping of single spines in cortical neurons in vivo. Nature 475, 501–505. 21706031 10.1038/nature10193

[tjp13069-bib-0017] Colbert CM , Magee JC , Hoffman DA & Johnston D (1997). Slow recovery from inactivation of Na+ channels underlies the activity‐dependent attenuation of dendritic action potentials in hippocampal CA1 pyramidal neurons. J Neurosci 17, 6512–6521. 9254663 10.1523/JNEUROSCI.17-17-06512.1997 PMC6573147

[tjp13069-bib-0018] Das A & Narayanan R (2015). Active dendrites mediate stratified gamma‐range coincidence detection in hippocampal model neurons. J Physiol 593, 3549–3576. 26018187 10.1113/JP270688 PMC4560584

[tjp13069-bib-0019] DeBello WM , McBride TJ , Nichols GS , Pannoni KE , Sanculi D & Totten DJ (2014). Input clustering and the microscale structure of local circuits. Front Neural Circuits 8, 112. 25309336 10.3389/fncir.2014.00112 PMC4162353

[tjp13069-bib-0020] Destexhe A , Rudolph M & Pare D (2003). The high‐conductance state of neocortical neurons in vivo. Nat Rev Neurosci 4, 739–751. 12951566 10.1038/nrn1198

[tjp13069-bib-0021] Domnisoru C & Tank DW (2016). Monosynaptic inputs to hippocampal place cell dendrites In EMBO Workshop on Dendritic Anatomy, Molecules and Function. Foundation for Research and Technology – Hellas (FORTH), Heraklion.

[tjp13069-bib-0022] Domnisoru C & Tank DW (2017). Spine imaging reveals direct synaptic inputs to CA1 neurons during navigation. In *Society for Neuroscience Annual Meeting 2017* , Washington, DC Program no. 252.13. 2017 Neuroscience Meeting Planner. http://www.abstractsonline.com/pp8/-!/4376/presentation/4954.

[tjp13069-bib-0023] Drion G , O'Leary T & Marder E (2015). Ion channel degeneracy enables robust and tunable neuronal firing rates. Proc Natl Acad Sci U S A 112, E5361–E5370. 26354124 10.1073/pnas.1516400112 PMC4586887

[tjp13069-bib-0024] Druckmann S , Feng L , Lee B , Yook C , Zhao T , Magee JC & Kim J (2014). Structured synaptic connectivity between hippocampal regions. Neuron 81, 629–640. 24412418 10.1016/j.neuron.2013.11.026

[tjp13069-bib-0025] Edelman GM & Gally JA (2001). Degeneracy and complexity in biological systems. Proc Natl Acad Sci U S A 98, 13763–13768. 11698650 10.1073/pnas.231499798 PMC61115

[tjp13069-bib-0026] Foster WR , Ungar LH & Schwaber JS (1993). Significance of conductances in Hodgkin‐Huxley models. J Neurophysiol 70, 2502–2518. 7509859 10.1152/jn.1993.70.6.2502

[tjp13069-bib-0027] Gasparini S & Magee JC (2006). State‐dependent dendritic computation in hippocampal CA1 pyramidal neurons. J Neurosci 26, 2088–2100. 16481442 10.1523/JNEUROSCI.4428-05.2006 PMC6674927

[tjp13069-bib-0028] Geisler C , Diba K , Pastalkova E , Mizuseki K , Royer S & Buzsaki G (2010). Temporal delays among place cells determine the frequency of population theta oscillations in the hippocampus. Proc Natl Acad Sci U S A 107, 7957–7962. 20375279 10.1073/pnas.0912478107 PMC2867922

[tjp13069-bib-0029] Golding NL , Mickus TJ , Katz Y , Kath WL & Spruston N (2005). Factors mediating powerful voltage attenuation along CA1 pyramidal neuron dendrites. J Physiol 568, 69–82. 16002454 10.1113/jphysiol.2005.086793 PMC1474764

[tjp13069-bib-0030] Golding NL & Spruston N (1998). Dendritic sodium spikes are variable triggers of axonal action potentials in hippocampal CA1 pyramidal neurons. Neuron 21, 1189–1200. 9856473 10.1016/s0896-6273(00)80635-2

[tjp13069-bib-0031] Goldman MS , Golowasch J , Marder E & Abbott LF (2001). Global structure, robustness, and modulation of neuronal models. J Neurosci 21, 5229–5238. 11438598 10.1523/JNEUROSCI.21-14-05229.2001 PMC6762863

[tjp13069-bib-0032] Govindarajan A , Israely I , Huang SY & Tonegawa S (2011). The dendritic branch is the preferred integrative unit for protein synthesis‐dependent LTP. Neuron 69, 132–146. 21220104 10.1016/j.neuron.2010.12.008 PMC3032443

[tjp13069-bib-0033] Grienberger C , Chen X & Konnerth A (2015). Dendritic function in vivo. Trends Neurosci 38, 45–54. 25432423 10.1016/j.tins.2014.11.002

[tjp13069-bib-0034] Grienberger C , Milstein AD , Bittner KC , Romani S & Magee JC (2017). Inhibitory suppression of heterogeneously tuned excitation enhances spatial coding in CA1 place cells. Nat Neurosci 20, 417–426. 28114296 10.1038/nn.4486

[tjp13069-bib-0035] Grosenick L , Marshel JH & Deisseroth K (2015). Closed‐loop and activity‐guided optogenetic control. Neuron 86, 106–139. 25856490 10.1016/j.neuron.2015.03.034 PMC4775736

[tjp13069-bib-0036] Harvey CD , Collman F , Dombeck DA & Tank DW (2009). Intracellular dynamics of hippocampal place cells during virtual navigation. Nature 461, 941–946. 19829374 10.1038/nature08499 PMC2771429

[tjp13069-bib-0037] Hill DN , Varga Z , Jia H , Sakmann B & Konnerth A (2013). Multibranch activity in basal and tuft dendrites during firing of layer 5 cortical neurons in vivo. Proc Natl Acad Sci U S A 110, 13618–13623. 23904480 10.1073/pnas.1312599110 PMC3746846

[tjp13069-bib-0038] Hoffman DA , Magee JC , Colbert CM & Johnston D (1997). K^+^ channel regulation of signal propagation in dendrites of hippocampal pyramidal neurons. Nature 387, 869–875. 9202119 10.1038/43119

[tjp13069-bib-0039] Jahr CE & Stevens CF (1990). Voltage dependence of NMDA‐activated macroscopic conductances predicted by single‐channel kinetics. J Neurosci 10, 3178–3182. 1697902 10.1523/JNEUROSCI.10-09-03178.1990 PMC6570236

[tjp13069-bib-0040] Jia H , Rochefort NL , Chen X & Konnerth A (2010). Dendritic organization of sensory input to cortical neurons in vivo. Nature 464, 1307–1312. 20428163 10.1038/nature08947

[tjp13069-bib-0041] Kjelstrup KB , Solstad T , Brun VH , Hafting T , Leutgeb S , Witter MP , Moser EI & Moser MB (2008). Finite scale of spatial representation in the hippocampus. Science 321, 140–143. 18599792 10.1126/science.1157086

[tjp13069-bib-0042] Klausberger T , Magill PJ , Marton LF , Roberts JD , Cobden PM , Buzsaki G & Somogyi P (2003). Brain‐state‐ and cell‐type‐specific firing of hippocampal interneurons in vivo. Nature 421, 844–848. 12594513 10.1038/nature01374

[tjp13069-bib-0043] Klausberger T & Somogyi P (2008). Neuronal diversity and temporal dynamics: the unity of hippocampal circuit operations. Science 321, 53–57. 18599766 10.1126/science.1149381 PMC4487503

[tjp13069-bib-0044] Kleindienst T , Winnubst J , Roth‐Alpermann C , Bonhoeffer T & Lohmann C (2011). Activity‐dependent clustering of functional synaptic inputs on developing hippocampal dendrites. Neuron 72, 1012–1024. 22196336 10.1016/j.neuron.2011.10.015

[tjp13069-bib-0045] Lee D , Lin BJ & Lee AK (2012). Hippocampal place fields emerge upon single‐cell manipulation of excitability during behavior. Science 337, 849–853. 22904011 10.1126/science.1221489

[tjp13069-bib-0046] Leonardo A (2005). Degenerate coding in neural systems. J Comp Physiol 191, 995–1010. 16252121 10.1007/s00359-005-0026-0

[tjp13069-bib-0047] Losonczy A & Magee JC (2006). Integrative properties of radial oblique dendrites in hippocampal CA1 pyramidal neurons. Neuron 50, 291–307. 16630839 10.1016/j.neuron.2006.03.016

[tjp13069-bib-0048] Losonczy A , Makara JK & Magee JC (2008). Compartmentalized dendritic plasticity and input feature storage in neurons. Nature 452, 436–441. 18368112 10.1038/nature06725

[tjp13069-bib-0049] Losonczy A , Zemelman BV , Vaziri A & Magee JC (2010). Network mechanisms of theta related neuronal activity in hippocampal CA1 pyramidal neurons. Nat Neurosci 13, 967–972. 20639875 10.1038/nn.2597 PMC2921679

[tjp13069-bib-0050] Magee JC (1998). Dendritic hyperpolarization‐activated currents modify the integrative properties of hippocampal CA1 pyramidal neurons. J Neurosci 18, 7613–7624. 9742133 10.1523/JNEUROSCI.18-19-07613.1998 PMC6793032

[tjp13069-bib-0051] Magee JC & Cook EP (2000). Somatic EPSP amplitude is independent of synapse location in hippocampal pyramidal neurons. Nat Neurosci 3, 895–903. 10966620 10.1038/78800

[tjp13069-bib-0052] Magee JC & Johnston D (1995). Characterization of single voltage‐gated Na^+^ and Ca^2+^ channels in apical dendrites of rat CA1 pyramidal neurons. J Physiol 487, 67–90. 7473260 10.1113/jphysiol.1995.sp020862 PMC1156600

[tjp13069-bib-0053] Magee JC & Johnston D (1997). A synaptically controlled, associative signal for Hebbian plasticity in hippocampal neurons. Science 275, 209–213. 8985013 10.1126/science.275.5297.209

[tjp13069-bib-0054] Makino H & Malinow R (2011). Compartmentalized versus global synaptic plasticity on dendrites controlled by experience. Neuron 72, 1001–1011. 22196335 10.1016/j.neuron.2011.09.036 PMC3310180

[tjp13069-bib-0055] Malik R , Dougherty KA , Parikh K , Byrne C & Johnston D (2016). Mapping the electrophysiological and morphological properties of CA1 pyramidal neurons along the longitudinal hippocampal axis. Hippocampus 26, 341–361. 26333017 10.1002/hipo.22526 PMC4760884

[tjp13069-bib-0056] Marder E & Taylor AL (2011). Multiple models to capture the variability in biological neurons and networks. Nat Neurosci 14, 133–138. 21270780 10.1038/nn.2735 PMC3686573

[tjp13069-bib-0057] Mehta MR , Barnes CA & McNaughton BL (1997). Experience‐dependent, asymmetric expansion of hippocampal place fields. Proc Natl Acad Sci U S A 94, 8918–8921. 9238078 10.1073/pnas.94.16.8918 PMC23195

[tjp13069-bib-0058] Mehta MR , Quirk MC & Wilson MA (2000). Experience‐dependent asymmetric shape of hippocampal receptive fields. Neuron 25, 707–715. 10774737 10.1016/s0896-6273(00)81072-7

[tjp13069-bib-0059] Mel BW (1993). Synaptic integration in an excitable dendritic tree. J Neurophysiol 70, 1086–1101. 8229160 10.1152/jn.1993.70.3.1086

[tjp13069-bib-0060] Migliore M , Hoffman DA , Magee JC & Johnston D (1999). Role of an A‐type K^+^ conductance in the back‐propagation of action potentials in the dendrites of hippocampal pyramidal neurons. J Comput Neurosci 7, 5–15. 10481998 10.1023/a:1008906225285

[tjp13069-bib-0061] Milstein AD , Bloss EB , Apostolides PF , Vaidya SP , Dilly GA , Zemelman BV & Magee JC (2015). Inhibitory gating of input comparison in the CA1 microcircuit. Neuron 87, 1274–1289. 26402609 10.1016/j.neuron.2015.08.025

[tjp13069-bib-0062] Mishra P & Narayanan R (2015). High‐conductance states and A‐type K^+^ channels are potential regulators of the conductance‐current balance triggered by HCN channels. J Neurophysiol 113, 23–43. 25231614 10.1152/jn.00601.2013

[tjp13069-bib-0063] Mittal D & Narayanan R (2018). Degeneracy in the robust expression of spectral selectivity, subthreshold oscillations and intrinsic excitability of entorhinal stellate cells. J Neurophysiol. doi: 10.1152/jn.00136.2018. [Epub ahead of print]. PMC6101195 29718802

[tjp13069-bib-0064] Mukunda CL & Narayanan R (2017). Degeneracy in the regulation of short‐term plasticity and synaptic filtering by presynaptic mechanisms. J Physiol 595, 2611–2637. 28026868 10.1113/JP273482 PMC5390884

[tjp13069-bib-0065] Nadkarni S , Bartol TM , Sejnowski TJ & Levine H (2010). Modelling vesicular release at hippocampal synapses. PLoS Comput Biol 6, e1000983. 21085682 10.1371/journal.pcbi.1000983 PMC2978677

[tjp13069-bib-0066] Nadkarni S , Bartol TM , Stevens CF , Sejnowski TJ & Levine H (2012). Short‐term plasticity constrains spatial organization of a hippocampal presynaptic terminal. Proc Natl Acad Sci U S A 109, 14657–14662. 22908295 10.1073/pnas.1211971109 PMC3437845

[tjp13069-bib-0067] Nakamura T , Barbara JG , Nakamura K & Ross WN (1999). Synergistic release of Ca^2+^ from IP3‐sensitive stores evoked by synaptic activation of mGluRs paired with backpropagating action potentials. Neuron 24, 727–737. 10595522 10.1016/s0896-6273(00)81125-3

[tjp13069-bib-0068] Narayanan R , Dougherty KJ & Johnston D (2010). Calcium store depletion induces persistent perisomatic increases in the functional density of h channels in hippocampal pyramidal neurons. Neuron 68, 921–935. 21145005 10.1016/j.neuron.2010.11.033 PMC3024579

[tjp13069-bib-0069] Narayanan R & Johnston D (2007). Long‐term potentiation in rat hippocampal neurons is accompanied by spatially widespread changes in intrinsic oscillatory dynamics and excitability. Neuron 56, 1061–1075. 18093527 10.1016/j.neuron.2007.10.033 PMC2430016

[tjp13069-bib-0070] Narayanan R & Johnston D (2008). The h channel mediates location dependence and plasticity of intrinsic phase response in rat hippocampal neurons. J Neurosci 28, 5846–5860. 18509046 10.1523/JNEUROSCI.0835-08.2008 PMC2612942

[tjp13069-bib-0071] Narayanan R & Johnston D (2010). The h current is a candidate mechanism for regulating the sliding modification threshold in a BCM‐like synaptic learning rule. J Neurophysiol 104, 1020–1033. 20554832 10.1152/jn.01129.2009 PMC2934916

[tjp13069-bib-0072] Narayanan R & Johnston D (2012). Functional maps within a single neuron. J Neurophysiol 108, 2343–2351. 22933729 10.1152/jn.00530.2012 PMC3545169

[tjp13069-bib-0073] Packer AM , Peterka DS , Hirtz JJ , Prakash R , Deisseroth K & Yuste R (2012). Two‐photon optogenetics of dendritic spines and neural circuits. Nat Methods 9, 1202–1205. 23142873 10.1038/nmeth.2249 PMC3518588

[tjp13069-bib-0074] Palmer LM , Shai AS , Reeve JE , Anderson HL , Paulsen O & Larkum ME (2014). NMDA spikes enhance action potential generation during sensory input. Nat Neurosci 17, 383–390. 24487231 10.1038/nn.3646

[tjp13069-bib-0075] Pan B & Zucker RS (2009). A general model of synaptic transmission and short‐term plasticity. Neuron 62, 539–554. 19477155 10.1016/j.neuron.2009.03.025 PMC3035647

[tjp13069-bib-0076] Poirazi P , Brannon T & Mel BW (2003a). Arithmetic of subthreshold synaptic summation in a model CA1 pyramidal cell. Neuron 37, 977–987. 12670426 10.1016/s0896-6273(03)00148-x

[tjp13069-bib-0077] Poirazi P , Brannon T & Mel BW (2003b). Pyramidal neuron as two‐layer neural network. Neuron 37, 989–999. 12670427 10.1016/s0896-6273(03)00149-1

[tjp13069-bib-0078] Prakriya M & Lewis RS (2015). Store‐operated calcium channels. Physiol Rev 95, 1383–1436. 26400989 10.1152/physrev.00020.2014 PMC4600950

[tjp13069-bib-0079] Prinz AA , Bucher D & Marder E (2004). Similar network activity from disparate circuit parameters. Nat Neurosci 7, 1345–1352. 15558066 10.1038/nn1352

[tjp13069-bib-0080] Pyapali GK , Sik A , Penttonen M , Buzsaki G & Turner DA (1998). Dendritic properties of hippocampal CA1 pyramidal neurons in the rat: intracellular staining in vivo and in vitro. J Comp Neurol 391, 335–352. 9492204 10.1002/(sici)1096-9861(19980216)391:3<335::aid-cne4>3.0.co;2-2

[tjp13069-bib-0081] Rall W (1977). Core conductor theory and cable properties of neurons In Handbook of Physiology. The Nervous System. Cellular Biology of Neurons, ed. Kandel ER, pp. 39–97. American Physiological Society, Bethesda, MD.

[tjp13069-bib-0082] Rathour RK & Narayanan R (2012a). Inactivating ion channels augment robustness of subthreshold intrinsic response dynamics to parametric variability in hippocampal model neurons. J Physiol 590, 5629–5652. 22930270 10.1113/jphysiol.2012.239418 PMC3528982

[tjp13069-bib-0083] Rathour RK & Narayanan R (2012b). Influence fields: a quantitative framework for representation and analysis of active dendrites. J Neurophysiol 107, 2313–2334. 22262825 10.1152/jn.00846.2011

[tjp13069-bib-0084] Rathour RK & Narayanan R (2014). Homeostasis of functional maps in active dendrites emerges in the absence of individual channelostasis. Proc Natl Acad Sci U S A 111, E1787–E1796. 24711394 10.1073/pnas.1316599111 PMC4035944

[tjp13069-bib-0085] Rathour RK & Narayanan R (2017). Degeneracy in hippocampal physiology and plasticity. bioRxiv 10.1101/203943. PMC6771840 31301166

[tjp13069-bib-0086] Regehr WG (2012). Short‐term presynaptic plasticity. Cold Spring Harb Perspect Biol 4, a005702. 22751149 10.1101/cshperspect.a005702 PMC3385958

[tjp13069-bib-0087] Regehr WG , Carey MR & Best AR (2009). Activity‐dependent regulation of synapses by retrograde messengers. Neuron 63, 154–170. 19640475 10.1016/j.neuron.2009.06.021 PMC3251517

[tjp13069-bib-0088] Ross WN (2012). Understanding calcium waves and sparks in central neurons. Nat Rev Neurosci 13, 157–168. 22314443 10.1038/nrn3168 PMC4501263

[tjp13069-bib-0089] Schiller J & Schiller Y (2001). NMDA receptor‐mediated dendritic spikes and coincident signal amplification. Curr Opin Neurobiol 11, 343–348. 11399433 10.1016/s0959-4388(00)00217-8

[tjp13069-bib-0090] Shah MM , Migliore M , Valencia I , Cooper EC & Brown DA (2008). Functional significance of axonal Kv7 channels in hippocampal pyramidal neurons. Proc Natl Acad Sci U S A 105, 7869–7874. 18515424 10.1073/pnas.0802805105 PMC2408483

[tjp13069-bib-0091] Sheffield ME & Dombeck DA (2015). Calcium transient prevalence across the dendritic arbour predicts place field properties. Nature 517, 200–204. 25363782 10.1038/nature13871 PMC4289090

[tjp13069-bib-0092] Shemesh OA , Tanese D , Zampini V , Linghu C , Piatkevich K , Ronzitti E , Papagiakoumou E , Boyden ES & Emiliani V (2017). Temporally precise single‐cell‐resolution optogenetics. Nat Neurosci 20, 1796–1806. 29184208 10.1038/s41593-017-0018-8 PMC5726564

[tjp13069-bib-0093] Sinha M & Narayanan R (2015). HCN channels enhance spike phase coherence and regulate the phase of spikes and LFPs in the theta‐frequency range. Proc Natl Acad Sci U S A 112, E2207–E2216. 25870302 10.1073/pnas.1419017112 PMC4418872

[tjp13069-bib-0094] Spruston N , Schiller Y , Stuart G & Sakmann B (1995). Activity‐dependent action potential invasion and calcium influx into hippocampal CA1 dendrites. Science 268, 297–300. 7716524 10.1126/science.7716524

[tjp13069-bib-0095] Srikanth S & Narayanan R (2015). Variability in state‐dependent plasticity of intrinsic properties during cell‐autonomous self‐regulation of calcium homeostasis in hippocampal model neurons. eNeuro 2, e0053‐0015.2015. 10.1523/ENEURO.0053-15.2015 PMC4596012 26464994

[tjp13069-bib-0096] Strange BA , Witter MP , Lein ES & Moser EI (2014). Functional organization of the hippocampal longitudinal axis. Nat Rev Neurosci 15, 655–669. 25234264 10.1038/nrn3785

[tjp13069-bib-0097] Stuart G & Spruston N (1998). Determinants of voltage attenuation in neocortical pyramidal neuron dendrites. J Neurosci 18, 3501–3510. 9570781 10.1523/JNEUROSCI.18-10-03501.1998 PMC6793161

[tjp13069-bib-0098] Takahashi N , Kitamura K , Matsuo N , Mayford M , Kano M , Matsuki N & Ikegaya Y (2012). Locally synchronized synaptic inputs. Science 335, 353–356. 22267814 10.1126/science.1210362

[tjp13069-bib-0099] Taylor AL , Goaillard JM & Marder E (2009). How multiple conductances determine electrophysiological properties in a multicompartment model. J Neurosci 29, 5573–5586. 19403824 10.1523/JNEUROSCI.4438-08.2009 PMC2821064

[tjp13069-bib-0100] Vaidya SP & Johnston D (2013). Temporal synchrony and gamma‐to‐theta power conversion in the dendrites of CA1 pyramidal neurons. Nat Neurosci 16, 1812–1820. 24185428 10.1038/nn.3562 PMC3958963

[tjp13069-bib-0101] Varga Z , Jia H , Sakmann B & Konnerth A (2011). Dendritic coding of multiple sensory inputs in single cortical neurons in vivo. Proc Natl Acad Sci U S A 108, 15420–15425. 21876170 10.1073/pnas.1112355108 PMC3174623

[tjp13069-bib-0102] Wang Y , Deng X , Mancarella S , Hendron E , Eguchi S , Soboloff J , Tang XD & Gill DL (2010). The calcium store sensor, STIM1, reciprocally controls Orai and CaV1.2 channels. Science 330, 105–109. 20929813 10.1126/science.1191086 PMC3601900

[tjp13069-bib-0103] Whitacre J & Bender A (2010). Degeneracy: a design principle for achieving robustness and evolvability. J Theor Biol 263, 143–153. 19925810 10.1016/j.jtbi.2009.11.008

[tjp13069-bib-0104] Whitacre JM (2010). Degeneracy: a link between evolvability, robustness and complexity in biological systems. Theor Biol Med Model 7, 6. 20167097 10.1186/1742-4682-7-6 PMC2830971

[tjp13069-bib-0105] Wilson DE , Whitney DE , Scholl B & Fitzpatrick D (2016). Orientation selectivity and the functional clustering of synaptic inputs in primary visual cortex. Nat Neurosci 19, 1003–1009. 27294510 10.1038/nn.4323 PMC5240628

